# Analysis of genomic and transcriptomic variations as prognostic signature for lung adenocarcinoma

**DOI:** 10.1186/s12859-020-03691-3

**Published:** 2020-09-30

**Authors:** Talip Zengin, Tuğba Önal-Süzek

**Affiliations:** 1 Department of Bioinformatics, Muğla Sıtkı Koçman University, Muğla, Turkey; 2 Department of Molecular Biology and Genetics, Muğla Sıtkı Koçman University, Muğla, Turkey; 3 Department of Computer Engineering, Muğla Sıtkı Koçman University, Muğla, Turkey

**Keywords:** TCGA, Lung cancer, Lung adenocarcinoma, Differential expression, SNV, CNV, Active subnetwork, Cox proportional hazards regression, Signature, Survival

## Abstract

**Background:**

Lung cancer is the leading cause of the largest number of deaths worldwide and lung adenocarcinoma is the most common form of lung cancer. In order to understand the molecular basis of lung adenocarcinoma, integrative analysis have been performed by using genomics, transcriptomics, epigenomics, proteomics and clinical data. Besides, molecular prognostic signatures have been generated for lung adenocarcinoma by using gene expression levels in tumor samples. However, we need signatures including different types of molecular data, even cohort or patient-based biomarkers which are the candidates of molecular targeting.

**Results:**

We built an R pipeline to carry out an integrated meta-analysis of the genomic alterations including single-nucleotide variations and the copy number variations, transcriptomics variations through RNA-seq and clinical data of patients with lung adenocarcinoma in The Cancer Genome Atlas project. We integrated significant genes including single-nucleotide variations or the copy number variations, differentially expressed genes and those in active subnetworks to construct a prognosis signature. Cox proportional hazards model with Lasso penalty and LOOCV was used to identify best gene signature among different gene categories.

We determined a 12-gene signature (BCHE, CCNA1, CYP24A1, DEPTOR, MASP2, MGLL, MYO1A, PODXL2, RAPGEF3, SGK2, TNNI2, ZBTB16) for prognostic risk prediction based on overall survival time of the patients with lung adenocarcinoma. The patients in both training and test data were clustered into high-risk and low-risk groups by using risk scores of the patients calculated based on selected gene signature. The overall survival probability of these risk groups was highly significantly different for both training and test datasets.

**Conclusions:**

This 12-gene signature could predict the prognostic risk of the patients with lung adenocarcinoma in TCGA and they are potential predictors for the survival-based risk clustering of the patients with lung adenocarcinoma. These genes can be used to cluster patients based on molecular nature and the best candidates of drugs for the patient clusters can be proposed. These genes also have a high potential for targeted cancer therapy of patients with lung adenocarcinoma.

## Background

Lung cancer is the most common cancer and responsible for the largest number of deaths worldwide with 1.8 million deaths, 18.4% of the total [1]. Lung cancer is categorized into two main categories: non-small cell lung cancer (NSCLC) which occurs in 85% of patients and small cell lung cancer (SCLC) in 15% of cases. NSCLC is grouped into 3 histological sub-types: lung adenocarcinoma (LUAD) which is most common form of lung cancer, lung squamous cell carcinoma (LUSC) and large cell carcinoma [2].

The integration of different types of molecular data has been used to characterize the molecular basis of lung cancer and to determine the clinical status of patients. Shi et al. analyzed 101 LUAD samples by using data from different levels -DNA mutations, gene expression profile, copy number variations and DNA methylation- in order to identify the relation between the genomic status and the clinical status. They determined deleterious mutations at ZKSCAN1 and POU4F2 genes which are two novel candidate driver genes [3]. Furthermore, recent studies have been performed to generate new methods to analyze integrative cancer data. Berger et al. proposed a new method called expression-based variant-impact phenotyping (eVIP) using differentially expressed genes (DEGs) to distinguish impactful from neutral somatic mutations. They characterized 194 somatic mutations related to primary LUAD and claimed that 69% of mutations were impactful. They determined the functionally important and actionable variants such as EGFR (p.S645C), ERBB2 (p.S418T), ARAF (p.S214C) and ARAF (p.S214F) although they are rare somatic mutations [4]. TCGA research network analyzed 230 LUAD samples using mRNA, microRNA and DNA sequencing integrated with copy number, methylation and proteomic data and reported the samples with high rates of somatic mutation [5]. Eighteen genes with high mutation load were reported such as RIT1 activating mutations and MGA loss-of-function mutations. They also identified aberrations in NF1, MET, ERBB2 and RIT1 occurred in 13% of cases and MAPK and PI(3)K pathway activity [5]. Deng et al. presented genomic alterations in LUAD samples from TCGA and found the significantly aberrant CNV segments which are associated with the immune system and 63 mutated genes associated with lung cancer signaling related to cancer progression. They identified important mutations of the PI3K protein family members include PIK3C2B, PIK3CA, PIK3R1 [6].

Recently, studies have been performed to generate gene signatures predicting prognosis risk of patients with lung adenocarcinoma. Krzystanek et al. identified a 7-gene signature by using microarray data of early-stage lung adenocarcinoma from GEO datasets. The genes (ADAM10, DLGAP5, RAD51AP1, FGFR10P, NCGAP, KIF15, ASPM) which have high hazards ratios showed significant results at cox regression analysis and Kaplan-Meier survival plots [7]. Shukla et al. identified 96 genes including five long noncoding RNAs (lncRNAs) among training data which had a prognostic association at test data, by using lung adenocarcinoma RNA-seq and clinical data from TCGA [8]. Shi et al. studied long noncoding RNAs (lncRNAs) expression signature model to predict stage I lung adenocarcinoma from TCGA and determined 31-lncRNA signature to predict overall survival in patients with LUAD [9]. Zhao et al. used gene expression profiles from TCGA and identified 20 genes that were significantly associated with the overall survival (OS). When they combined with GEO data set, they obtained four genes, FUT4, SLC25A42, IGFBP1, and KLHDC8B as common [10]. Li et al. performed RNA-sequencing on LUAD tumor samples and normal tissue samples. They construct protein–protein interaction network by using DEGs which were the intersection of GEO datasets and identified hub genes. Then, they test these genes on patient cohorts and TCGA data. They identified eight genes (DLGAP5, KIF11, RAD51AP1, CCNB1, AURKA, CDC6, OIP5 and NCAPG) which were closely related to survival in LUAD [11]. He et al. studied on previous GEO datasets and TCGA data and they identified a 8-gene prognostic signature (CDCP1, HMMR, TPX2, CIRBP, HLF, KBTBD7, SEC24B-AS1, and SH2B1) by using the step-wise multivariate Cox analysis. These genes were good predictors of survival between the high-risk and low-risk groups of patients with early-stage NSCLC [12]. The studies above determined different gene signatures for prognosis risk prediction by using different methods and presented different genes. Although, mostly gene expression data has been used for this purpose, we integrated SNVs, CNVs, DEGs and active subnetwork DEGs to generate gene signature for risk model by using LUAD data from The Cancer Genome Atlas (TCGA) database which provides simple nucleotide variation, gene expression, miRNA expression, DNA methylation, copy number variation and reverse phase protein array, clinical and biospecimen data from more than 10,000 cancer patients with 39 cancer types [13].

**Fig. 1:**
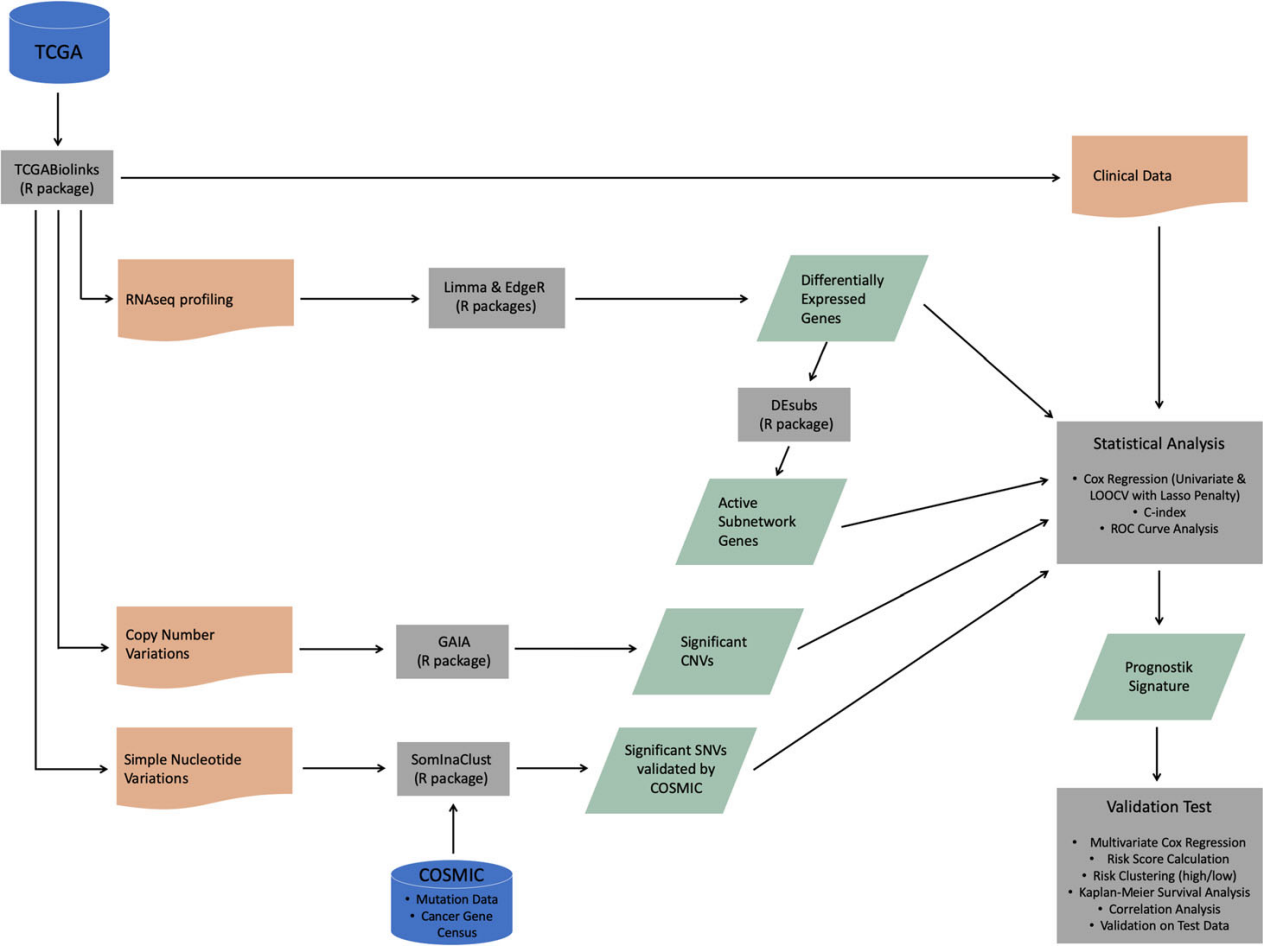
The R pipeline used for construction and validation of the prognosis gene signature

In this study, we built an R pipeline (Fig. 1) to perform an integrative analysis including SNVs and CNVs, differentially expressed genes and clinical data of patients with lung adenocarcinoma in TCGA. We generated different data categories by using significant SNVs, CNVs, DEGs and active subnetwork DEGs. Multivariate Cox proportional hazards model with the Lasso penalty and LOOCV was used to identify best gene signature among different gene categories. We generated 12-gene signature (BCHE, CCNA1, CYP24A1, DEPTOR, MASP2, MGLL, MYO1A, PODXL2, RAPGEF3, SGK2, TNNI2, ZBTB16) for prognostic risk prediction based on overall survival time of the patients with lung adenocarcinoma. When we clustered patients into high-risk and low-risk groups, the survival analysis showed highly significant results for both training and test datasets.

## Results

### Identification of significant simple nucleotide variations

**Fig. 2:**
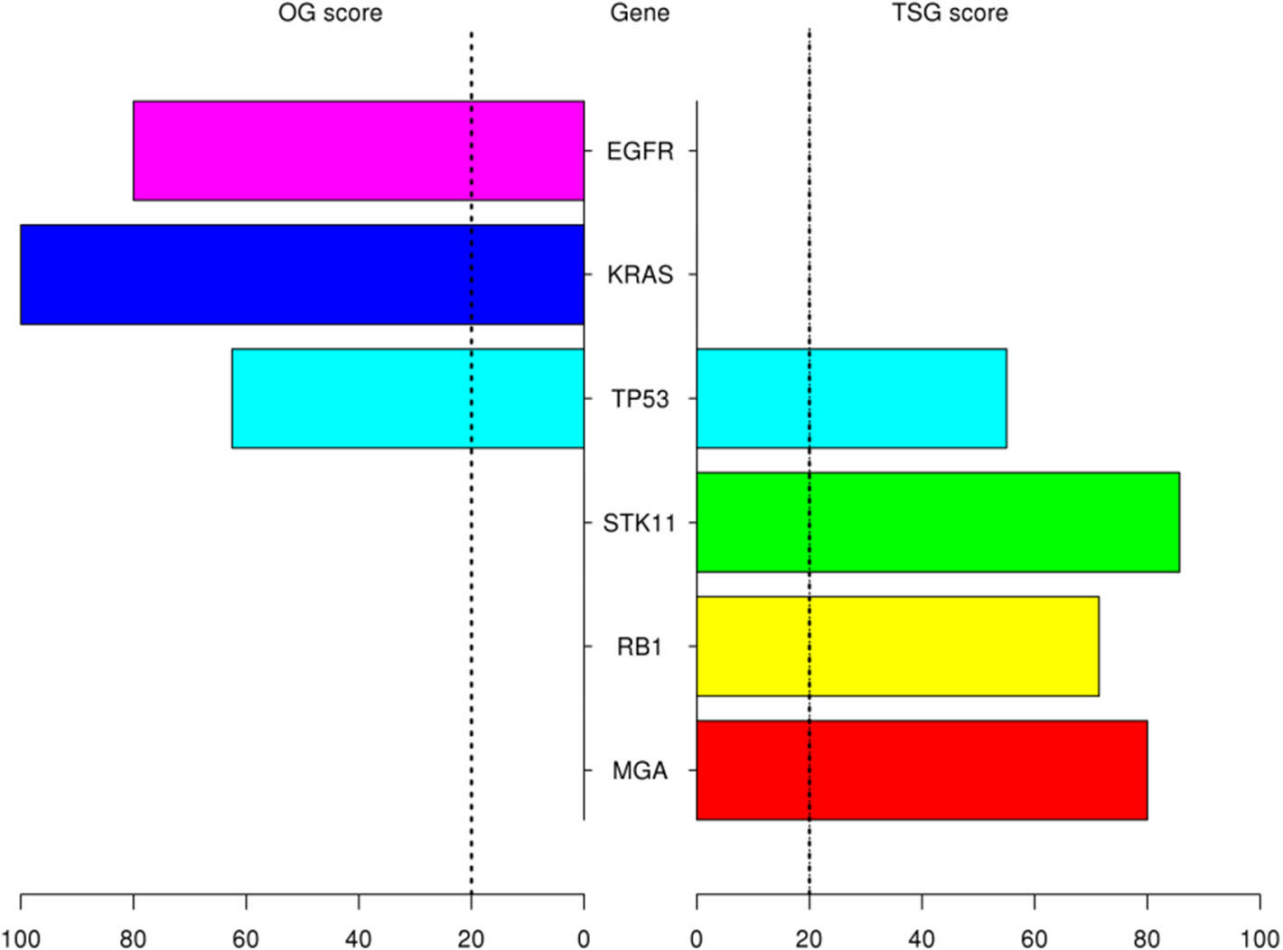
Pyramid plot of the significantly mutated genes in tumor samples of 55 patients with LUAD. The oncogene (OG) or tumor suppressor gene (TSG) scores were calculated by *SomInaClust* R package

Mutation data of LUAD patients as maf file generated by mutect pipeline was downloaded by *TCGAbiolinks* package and *maftools* package was used to subset original maf file by tumor sample barcodes of 55 LUAD patients (who have paired RNAseq data) and 510 LUAD patients (all patients in LUAD project who have all types of data used in the study). Then, significant mutations for both 55 and 510 LUAD patients were determined separately with their roles as a tumor suppressor or an oncogene by *SomInaClust* R package. In order to determine important genes including significant mutation clusters, we used *SomInaClust* R package. EGFR, KRAS, TP53, STK11, RB1 and MGA genes were determined as candidate driver genes in tumor samples of 55 LUAD patients (Fig. 2). EGFR and KRAS genes were classified as oncogenes and STK11, RB1 and MGA genes were classified as tumor suppressors. Although TP53 gene has both OG score and TSG score, TP53 was classified as a tumor suppressor in Table 1 depending on reference information of the cancer gene census. EGFR, KRAS, TP53, STK11 and RB1 have highly significant estimation. While EGFR and TP53 have high number of mutations, KRAS, STK11, RB1 and MGA have low number of mutations. While EGFR, KRAS, TP53, STK11, RB1 are well known cancer related genes, MGA gene is not in the cancer gene census.

**Table 1:** Significantly mutated genes in tumor samples of 55 patients with LUAD. The genes were classified as an oncogene (OG) and a tumor suppressor gene (TSG) based on their scores and the cancer gene census information by *SomInaClust* R package

Gene	# Mutations	Q value	OG Score	TSG Score	Classification	CGC^**a**^
EGFR	11	1.57e-12	80	0	OG	Dom
KRAS	8	1.57e-12	100	0	OG	Dom
TP53	20	4.8e-07	62.5	55	TSG	Rec
STK11	7	0.000106	0	85.7	TSG	Rec
RB1	7	0.0049	0	71.4	TSG	Rec
MGA	6	0.0217	0	80	TSG	NA

^a^Cancer gene census (*Dom* Dominant, *Rec* Recessive)

**Fig. 3:**
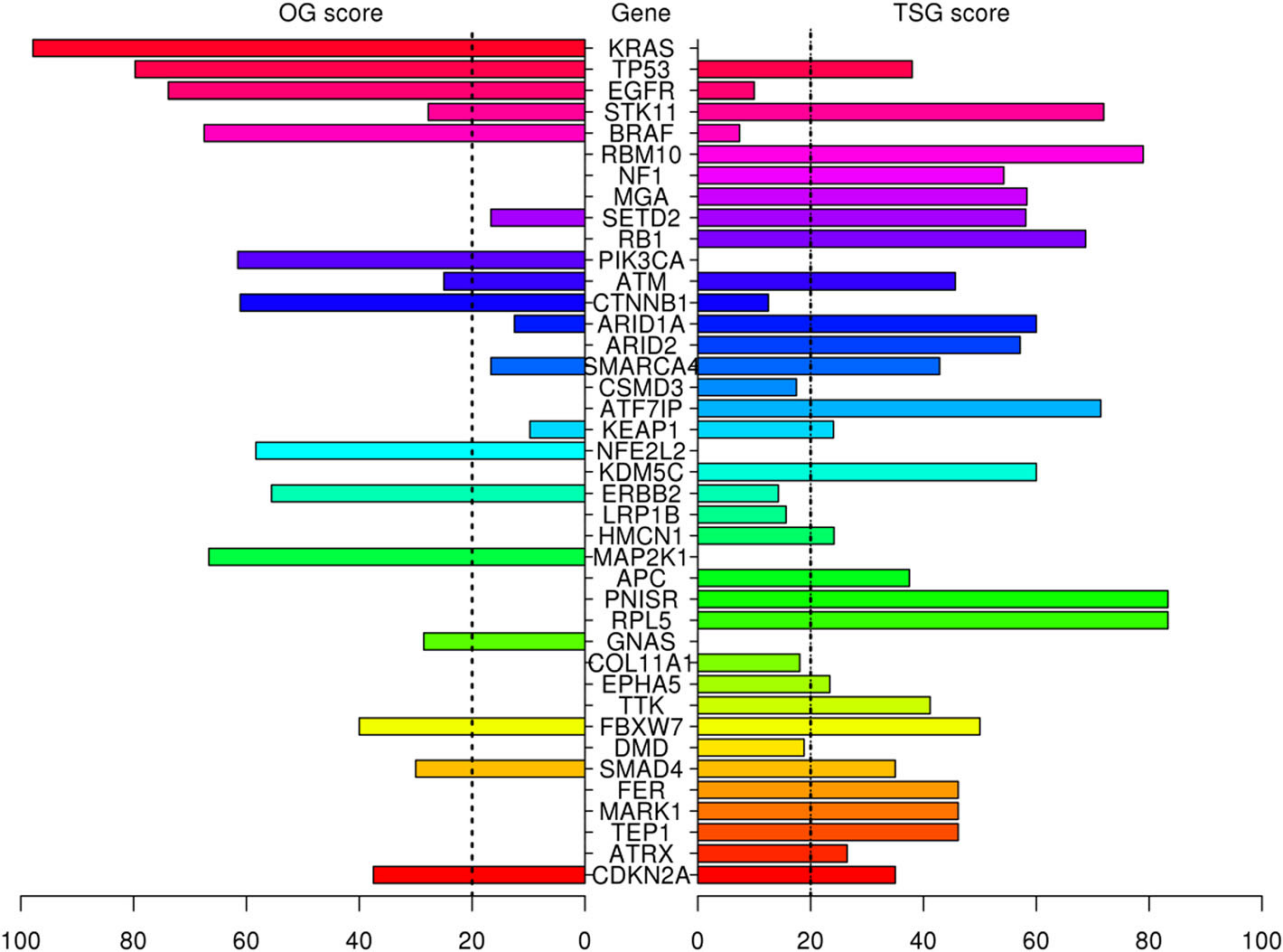
Pyramid plot of the top 40 significantly mutated genes in tumor samples of 510 patients with LUAD. The oncogene (OG) and tumor suppressor gene (TSG) scores were calculated by *SomInaClust* R package

Eighty-two genes were identified as candidate driver genes in tumor samples of 510 LUAD patients (Table 2), including KRAS, TP53, EGFR, STK11, MGA and RB1 which were determined also in tumor samples of 55 LUAD patients (Fig. 3). These genes include very well-known cancer related oncogenes such as BRAF, ERBB2, AKT1 and PIK3CA with the genes which are not listed in the cancer gene census list of the COSMIC database (Table 2).

**Table 2:** Significantly mutated genes in tumor samples of 510 patients with LUAD. The genes were classified as oncogene (OG) and tumor suppressor gene (TSG) based on their scores and the cancer gene census information by *SomInaClust* R package

Gene	# Mutations	qDG	OG Score	TSG Score	Classification	CGC^**a**^
KRAS	143	1.97e-250	97.8	0	OG	Dom
TP53	253	2.52e-135	79.7	38	TSG	Rec
EGFR	73	8.97e-84	73.8	10	OG	Dom
STK11	83	4.6e-61	27.8	72	TSG	Rec
BRAF	44	8.07e-51	67.5	7.4	OG	Dom
RBM10	39	9.06e-31	0	78.9	TSG	NA
NF1	63	5.37e-25	0	54.2	TSG	Rec
MGA	52	6.46e-23	0	58.3	TSG	NA
SETD2	44	1.34e-20	16.7	58.1	TSG	Rec
RB1	32	4.99e-20	0	68.8	TSG	Rec
PIK3CA	27	1.36e-19	61.5	0	OG	Dom
ATM	48	5.18e-18	25	45.7	TSG	Rec
CTNNB1	21	3.32e-15	61.1	12.5	OG	Dom
ARID1A	30	1.76e-14	12.5	60	TSG	Rec
ARID2	29	2.83e-12	0	57.1	TSG	Rec
SMARCA4	48	2.23e-11	16.7	42.9	TSG	Rec
CSMD3	324	6.25e-10	0	17.5	NA	NA
ATF7IP	17	1.84e-08	0	71.4	TSG	NA
KEAP1	90	1.91e-08	9.8	24.1	TSG	NA
NFE2L2	14	2.83e-07	58.3	0	OG	Dom
KDM5C	16	1.76e-06	0	60	TSG	Rec
ERBB2	13	6.94e-06	55.6	14.3	OG	Dom
LRP1B	267	6.04e-05	0	15.6	NA	Rec
HMCN1	97	8.93e-05	0	24.1	TSG	NA
MAP2K1	9	0.000263	66.7	0	OG	Dom
APC	24	0.000272	0	37.5	TSG	Rec
PNISR	6	0.000626	0	83.3	TSG	NA
RPL5	7	0.000626	0	83.3	TSG	Dom
GNAS	19	0.000962	28.6	0	OG	Dom
COL11A1	129	0.00139	0	18.1	NA	NA
EPHA5	66	0.00221	0	23.4	TSG	NA
TTK	18	0.00221	0	41.2	TSG	NA
FBXW7	12	0.0028	40	50	TSG	Rec
DMD	99	0.00349	0	18.8	NA	NA
SMAD4	20	0.00379	30	35	TSG	Rec
FER	16	0.0043	0	46.2	TSG	NA
MARK1	21	0.0043	0	46.2	TSG	NA
TEP1	29	0.0043	0	46.2	TSG	NA
ATRX	35	0.00463	0	26.5	TSG	Rec
CDKN2A	21	0.00585	37.5	35	TSG	Rec
MYO9A	19	0.00615	0	42.9	TSG	NA
ZNF800	17	0.00615	0	42.9	TSG	NA
CMTR2	26	0.00674	0	55.6	TSG	NA
RASA1	9	0.00674	0	55.6	TSG	NA
CDKN1B	5	0.00674	0	80	TSG	Rec
DHX15	7	0.00674	0	80	TSG	NA
IQGAP2	28	0.00816	0	40	TSG	NA
LTN1	19	0.00816	0	40	TSG	NA
SMARCA1	19	0.00816	0	40	TSG	NA
SPTA1	164	0.00971	0	17.6	NA	NA
FHOD3	31	0.0122	0	30.4	TSG	NA
CPVL	8	0.0161	0	66.7	TSG	NA
MAP3K12	8	0.0161	0	66.7	TSG	NA
TOP2B	9	0.0161	0	66.7	TSG	NA
ROCK1	21	0.0163	0	35.3	TSG	NA
PBRM1	12	0.0172	0	45.5	TSG	Rec
AKAP6	40	0.0195	0	28	TSG	NA
SENP1	3	0.0241	0	100	TSG	NA
SP1	4	0.0241	0	100	TSG	NA
WISP3	4	0.0241	0	100	TSG	NA
RAD50	13	0.0243	20	41.7	TSG	NA
COL28A1	19	0.0243	0	41.7	TSG	NA
SCAF8	18	0.0243	0	41.7	TSG	NA
STK31	19	0.0243	0	41.7	TSG	NA
IDH1	6	0.0248	40	25	TSG	Dom
USH2A	240	0.0263	0	13.2	NA	NA
YLPM1	23	0.0269	0	31.6	TSG	NA
IQUB	12	0.0272	0	57.1	TSG	NA
MARK2	10	0.0272	0	57.1	TSG	NA
NAA15	8	0.0272	0	57.1	TSG	NA
CDH10	99	0.028	0	16.4	NA	NA
AKT1	3	0.0296	66.7	0	OG	Dom
RAF1	7	0.031	66.7	0	OG	Dom
VPS13C	39	0.0332	0	25	TSG	NA
ZBBX	28	0.0333	0	30	TSG	NA
DST	67	0.0333	0	19.1	NA	NA
KMT2C	52	0.0388	0	18.8	NA	Rec
DGKB	38	0.0431	0	28.6	TSG	NA
MAP2K4	8	0.045	33.3	50	TSG	Rec
FBN2	93	0.045	0	20.5	TSG	NA
B2M	8	0.045	0	50	TSG	Rec
BAP1	8	0.045	0	50	TSG	Rec

^a^Cancer gene census (*Dom* Dominant, *Rec* Recessive)

### Identification of the significant copy number variations

**Fig. 4:**
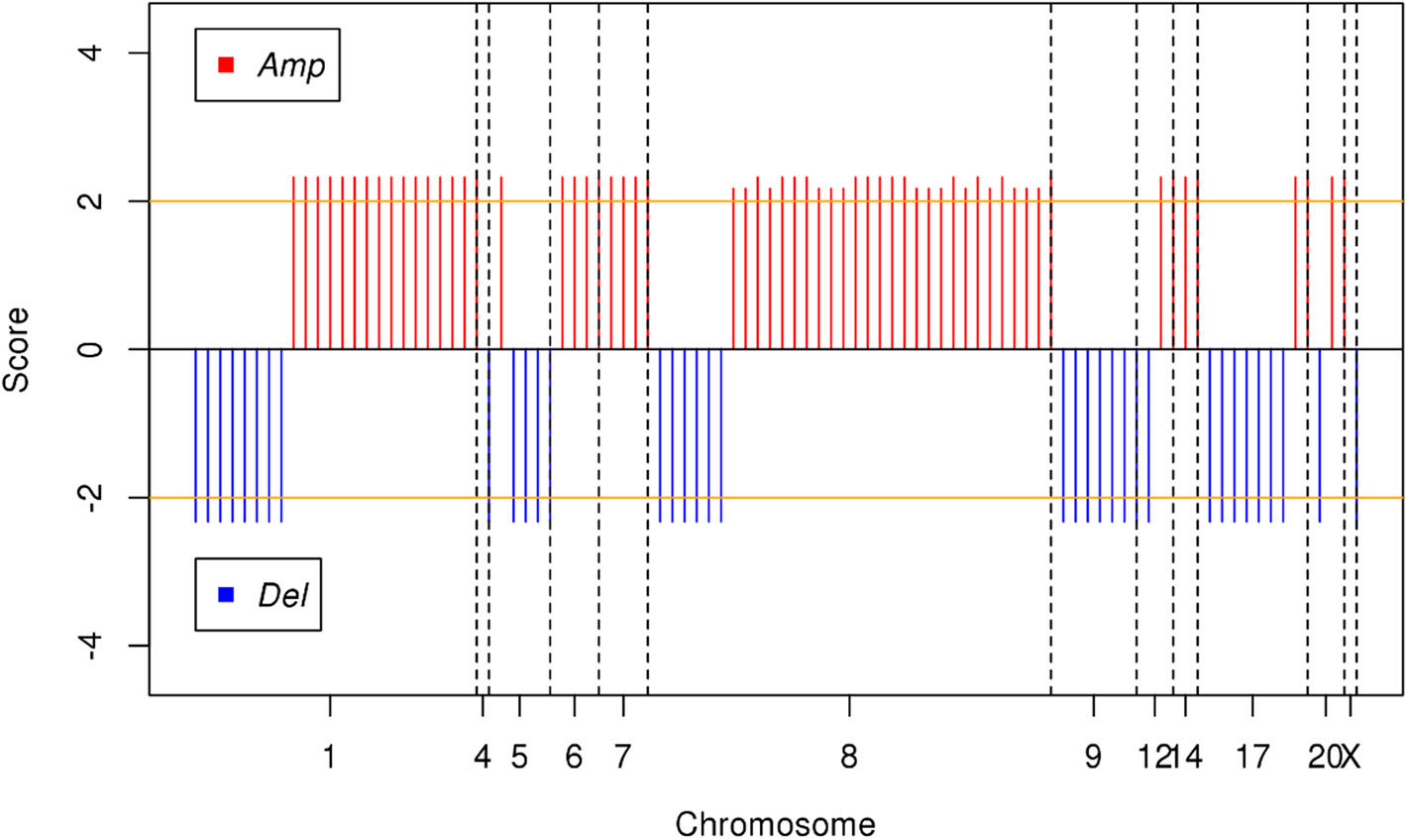
Significant CNVs on all chromosomes in tumor samples of 55 patients with LUAD. Orange line shows the q-value threshold (0.01) in -log_10_ base. Red lines which have positive scores shows the amplification of genomic regions on specified chromosome; blue lines which have negative scores shows the deletion of genomic regions on specified chromosome. The genomic regions above threshold were selected for further gene enrichment analysis. Amp: Amplification, Del: Deletion

CNVs (Copy Number Variations) are important aberrations which results alterations in gene expression in tumorigenesis and tumor growth. In order to determine the significant CNVs among tumor samples of 55 and 510 LUAD patients, *gaia* R package was used. Significant recurrent CNVs in tumor samples of 55 LUAD patients, over the q-value thresholds (0.01), are mostly observed on Chromosome 1, 8, 9, and 17. Chromosome 1 has the highest number of amplifications followed by Chromosome 8. Chromosome 9 has the highest number of deletions followed by Chromosome 17 as seen in Fig. 4. Chromosome 1 has the highest number of gene aberration with 2006 amplified or deleted genes followed by Chromosome 8 with 1029 aberrant genes and Chromosome 19 with 785 aberrant genes. Top ten significantly amplified and deleted genes which are all from chromosome 1 are listed in Table 3.

**Table 3:** Top ten significant deleted and amplified genes in tumor samples of 55 patients with LUAD. After gene enrichment analysis, the significantly amplified and deleted genes were listed with their aberration type, q value, genomic aberration region and gene region on specified chromosomes

Gene Symbol	Aberration	q-value	Aberrant Region	Gene Region
RN7SKP285	Del	0.00474651	1:103501576–107318961	1:103523562–10352879
RNPC3	Del	0.00474651	1:103501576–107318961	1:103525691–103555239
AMY2B	Del	0.00474651	1:103501576–10318961	1:103553815–103579534
ACTG1P4	Del	0.00474651	1:103501576–10731961	1:103569553–103570674
AMY2A	Del	0.00474651	1:103501576–107318961	1:103616811–103625780
AMY1A	Del	0.00474651	1:103501576–107318961	1:103655290–103664554
AC105272.1	Del	0.00474651	1:103501576–107318961	1:103668071–10668268
AMY1B	Del	0.00474651	1:103501576–107318961	1:103687415–103,696,680
AMYP1	Del	0.00474651	1:103501576–107318961	1:103713723–103719871
AMY1C	Del	0.00474651	1:103501576–107318961	1:103750406–103758690
PLEKHO1	Amp	0.00474651	1:150131878–150768299	1:150149183–150164720
AC242988.2	Amp	0.00474651	1:150131878–150768299	1:150173049–150181429
RN7SL480P	Amp	0.00474651	1:150131878–15768299	1:150211632–150211925
ANP32E	Amp	0.00474651	1:150131878–150768299	1:150218417–150236156
RNU2-17P	Amp	0.00474651	1:150131878–150768299	1:150236967–150237156
AC242988.1	Amp	0.00474651	1:150131878–150768299	1:150255095–150257286
CA14	Amp	0.00474651	1:150131878–15768299	1:150257251–150265078
APH1A	Amp	0.00474651	1:150131878–150768299	1:150265399–150269580
C1orf54	Amp	0.00474651	1:150131878–150768299	1:150268200–150280916
CIART	Amp	0.00474651	1:150131878–150768299	1:150282543–150287093

*Amp* Amplification, *Del* Deletion

**Fig. 5:**
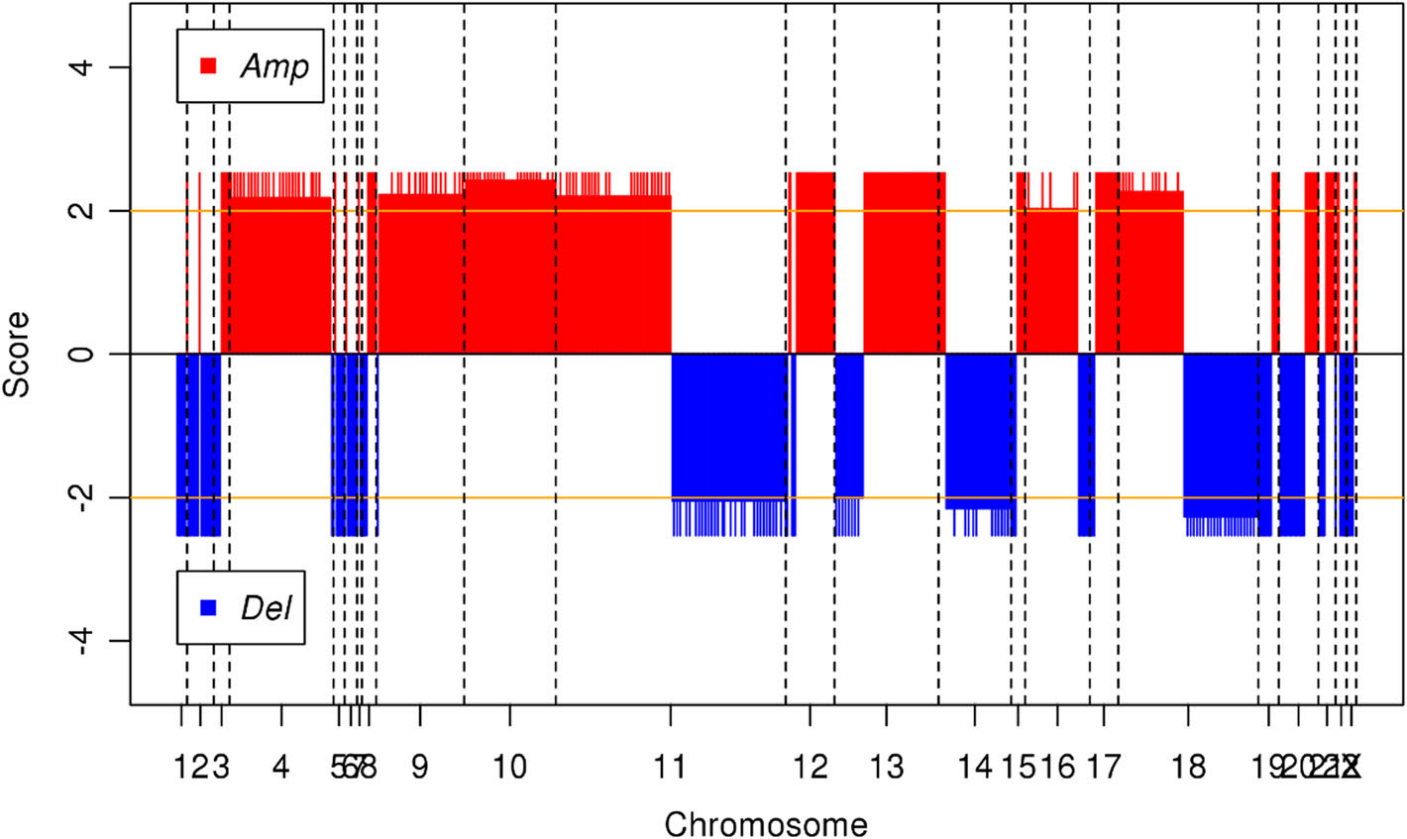
Significant CNVs on all chromosomes in tumor samples of 510 patients with LUAD. Orange line shows the q-value threshold (0.01) in -log_10_ base. Red lines which have positive scores shows the amplification of genomic regions on specified chromosome; blue lines which have negative scores shows the deletion of genomic regions on specified chromosome. The genomic regions above threshold were selected for further gene enrichment analysis. Amp: Amplification, Del: Deletion

Significant recurrent CNVs in tumor samples of 510 LUAD patients, over the q-value thresholds (0.01), are mostly observed on Chromosome 4, 9, 10, 11, 12, 13, 14, 16, 18 and 20. But Chromosome 11 has the highest number of aberrations followed by Chromosome 9, 16 and 18. Chromosome 4, 9, 10, 12 and 16 had mostly amplifications (Fig. 5). The pattern of CNVs in tumor samples of 510 patients has a marked difference from the CNV pattern in tumor samples of 55 patients (Fig. 4). Chromosome 1 has the highest number of gene aberration with 3124 amplified or deleted genes followed by Chromosome 6 with 2911 aberrant genes and Chromosome 3 with 2149 aberrant genes. Top ten significantly amplified and deleted genes which are all from chromosome 1 are shown in Table 4.

**Table 4:** Top ten significantly deleted and amplified genes in tumor samples of 510 patients with LUAD. After gene enrichment analysis, the significantly amplified and deleted genes were listed with their aberration type, q value, genomic aberration region and gene region on specified chromosomes

Gene Symbol	Aberration	q-value	Aberrant Region	Gene Region
AL359821.1	Del	0.0029609	1:71621685–71778398	1:71738173–71738354
GDI2P2	Del	0.0029609	1:71928758–119984738	1:72274552–72275159
AL513166.2	Del	0.0029609	1:71928758–119984738	1:72283170–72753772
RPL31P12	Del	0.0029609	1:71928758–119984738	1:72301472–72301829
AL583808.1	Del	0.0029609	1:71928758–119984738	1:72636547–72899240
RNU6-1246P	Del	0.0029609	1:71928758–119984738	1:72717663–72717769
AL583808.2	Del	0.0029609	1:71928758–119984738	1:72765031–72791282
AL583808.3	Del	0.0029609	1:71928758–119984738	1:72793104–72854475
AL732618.1	Del	0.0029609	1:71928758–119984738	1:72979014–72979314
KRT8P21	Del	0.0029609	1:71928758–119984738	1:73104792–73106282
SF3B4	Amp	0.0029609	1:149907993–247650984	1:149923317–149927803
MTMR11	Amp	0.0029609	1:149907993–247650984	1:149928651–149936879
OTUD7B	Amp	0.0029609	1:149907993–247650984	1:149937812–150010726
AC244033.2	Amp	0.0029609	1:149907993–247650984	1:150045660–150067701
AC244033.1	Amp	0.0029609	1:149907993–247650984	1:150053864–150055034
VPS45	Amp	0.0029609	1:149907993–247650984	1:150067279–150145329
PLEKHO1	Amp	0.0029609	1:149907993–247650984	1:150149183–150164720
AC242988.2	Amp	0.0029609	1:149907993–247650984	1:150173049–150181429
RN7SL480P	Amp	0.0029609	1:149907993–247650984	1:150211632–150211925
ANP32E	Amp	0.0029609	1:149907993–247650984	1:150218417–150236156

*Amp* Amplification, *Del* Deletion

### Differential expression analysis (DEA)

**Fig. 6:**
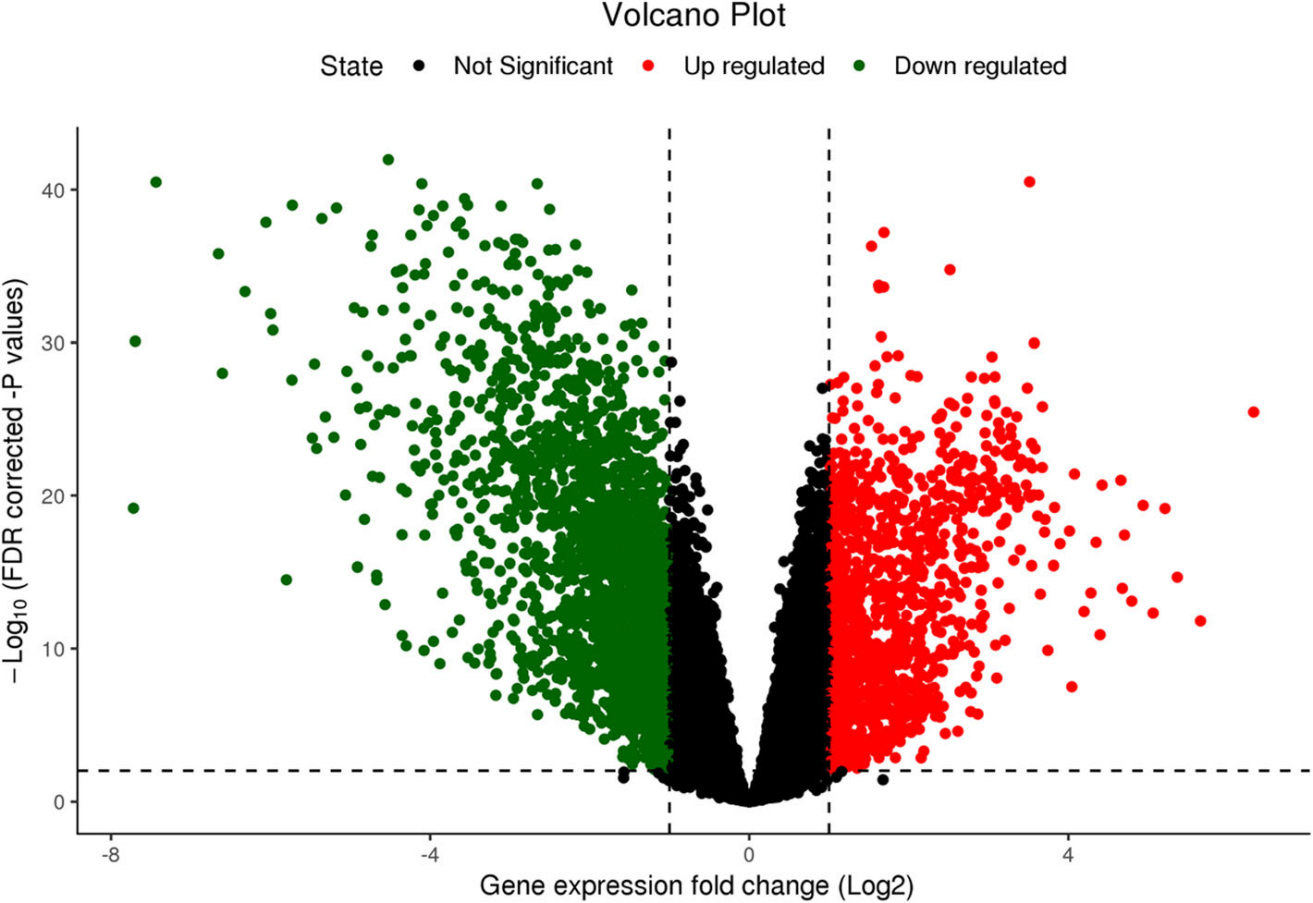
Volcano plot of differentially expressed genes in tumor samples of 55 patients with LUAD. Red dots represent up regulated genes more than two-fold significantly (q-value < 0.01) and green dots represent down regulated genes more than two-fold (log_2_=1) significantly (q-value < 0.01). The black dots represent the genes which have differential expression less than two-fold and/or not significant

The Transcriptome Profiling data of LUAD patients in mRNA expression level (as unnormalized *HTSeq* raw counts), was downloaded by *TCGABiolinks* R package. Differentially expressed genes were determined with FDR adjusted *p*-values (q-values) in tumor samples (TP) of 55 patients with LUAD compared to normal samples (NT) of the same patients by the *limma-voom* method using *limma* and *edgeR* R packages. The volcano plot in Fig. 6, shows the differentially expressed genes (DEGs) as dots of which black ones represent the genes which have differential expression less than two-fold and not significant while red ones represent upregulated and green ones downregulated more than two-fold (log_2_ = 1) significantly (q value < 0.01). As a result of this analysis, 3575 genes were dysregulated more than two-fold with 0.01 q-value significance.

As the result of DEA, differentially expressed genes (DEGs) are determined with their log Fold Change (logFC), adjusted p-value (q-value), entrez gene IDs and HGNC symbols after enrichment analysis. The top 10 down-regulated and up-regulated genes are shown in Tables 5 and 6. The list of DEGs was used for pathway analysis and active subnetwork analysis.

**Table 5:** Top ten significantly down-regulated genes in tumor samples of 55 patients with LUAD. After gene enrichment analysis by using Ensembl gene ID of DEGs, down-regulated genes were listed with their entrez gene ID, HGNC symbol, log_2_ fold change value and q-value (adj.P.Val)

ensembl_gene_id	entrezgene	hgnc_symbol	logFC	adj.P.Val
ENSG00000182010	219790	RTKN2	−4.52455117194123	1.07397390772473e-42
ENSG00000158764	142683	ITLN2	−7.4364942528429	3.19924465283634e-41
ENSG00000102683	6445	SGCG	−4.10485571819757	4.07515928515459e-41
ENSG00000198873	2869	GRK5	−2.65790712992412	4.07515928515459e-41
ENSG00000107742	9806	SPOCK2	−3.56967403596283	3.85300139768808e-40
ENSG00000170323	2167	FABP4	−5.72790493543673	1.03033381509032e-39
ENSG00000135063	9413	FAM189A2	−3.53046742312343	1.03117504787973e-39
ENSG00000186994	256949	KANK3	−3.1101996380779	1.15468325581686e-39
ENSG00000150625	2823	GPM6A	−5.17438700689996	1.5648953870669e-39
ENSG00000154721	58494	JAM2	−2.50261146610761	1.92231892168565e-39

**Table 6:** Top ten significantly up-regulated genes in tumor samples of 55 patients with LUAD. After gene enrichment analysis by using Ensembl gene ID of DEGs, up-regulated genes were listed with their entrez gene ID, HGNC symbol, log_2_ fold change value and q-value (adj.P.Val)

ensembl_gene_id	entrezgene	hgnc_symbol	logFC	adj.P.Val
ENSG00000183010	5831	PYCR1	3.5139225242735	3.06017765569688e-41
ENSG00000059573	5832	ALDH18A1	1.68852856318992	6.30895314373162e-38
ENSG00000164466	94081	SFXN1	1.5322079314688	5.01920971916517e-37
ENSG00000135052	51280	GOLM1	2.51608337184892	1.73125209540521e-35
ENSG00000180198	1104	RCC1	1.62119814668367	1.82637777402036e-34
ENSG00000155660	9601	PDIA4	1.6848754492746	2.37855372052335e-34
ENSG00000096063	6732	SRPK1	1.62823462104507	2.66740561460568e-34
ENSG00000128050	10606	PAICS	1.65390171937903	4.22169230063646e-31
ENSG00000111344	8437	RASAL1	3.57173273242386	1.08251787193746e-30
ENSG00000173457	26472	PPP1R14B	1.86684316566064	7.07845976872399e-30

### Active subnetwork and pathway analysis

**Fig. 7:**
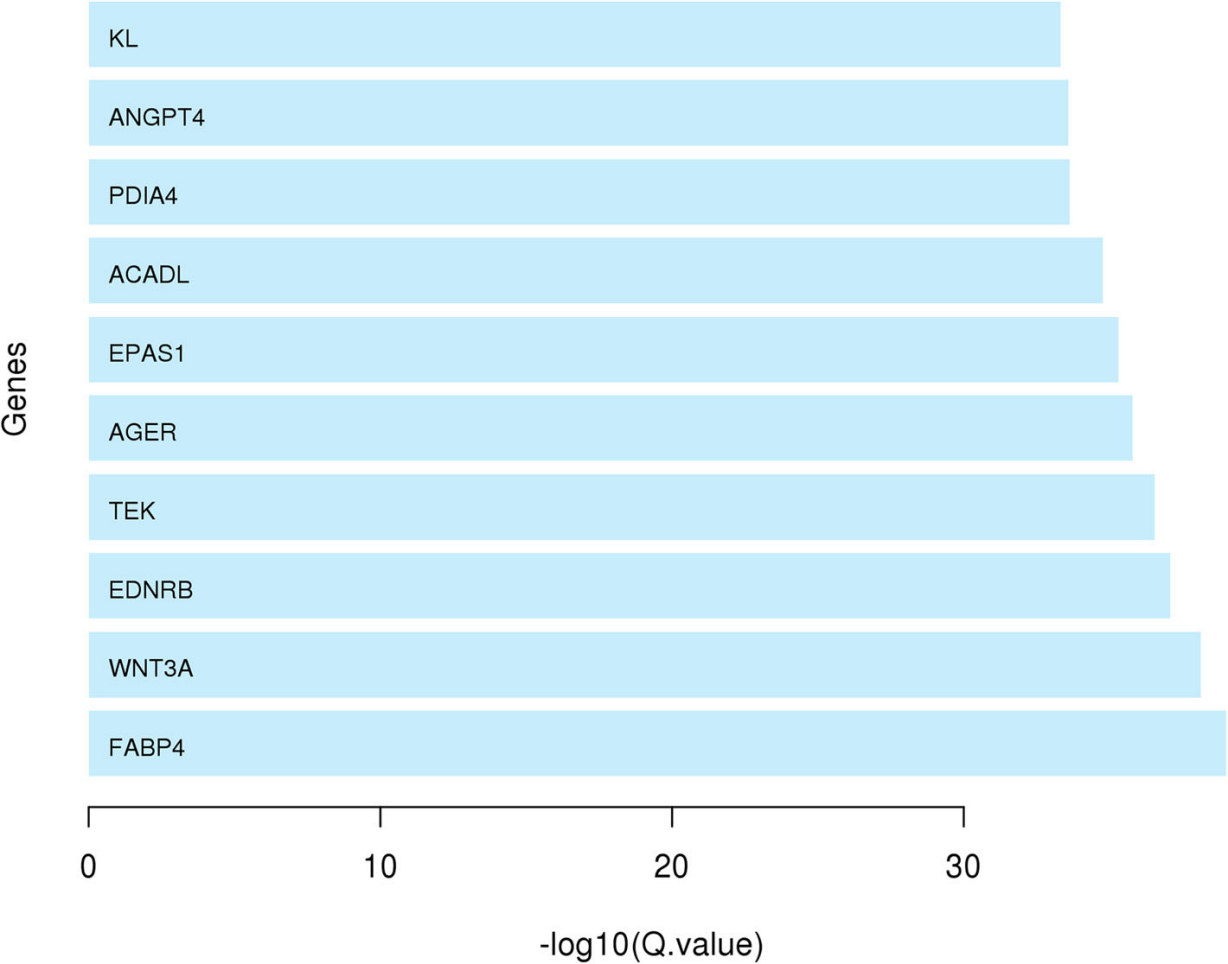
Top 10 significant active subnetwork genes in tumor samples of 55 LUAD patients. The values on x-axis showed the -log_10_(q-value), thus higher value means higher significance level

**Fig. 8:**
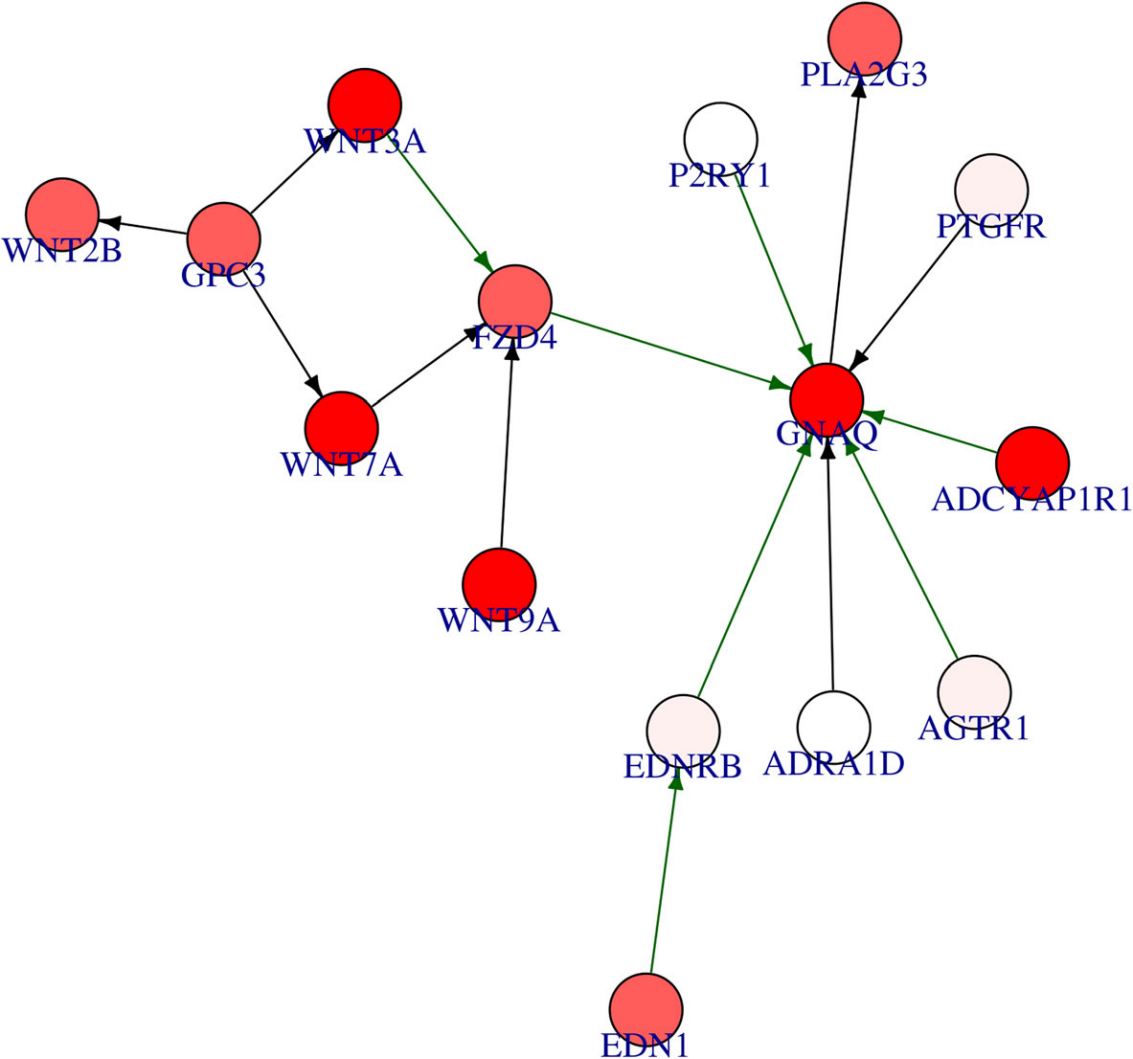
First active subnetwork from PPI network in tumor samples of 55 patients with LUAD. *DEsubs* R package identified active subnetworks by using q-values of DEGs determined at previous step. Red graduation in nodes indicate the q-value degree, the edge width indicates the correlation degree between the respective genes. Green or red color in edges indicates the positive or negative correlation respectively

**Fig. 9:**
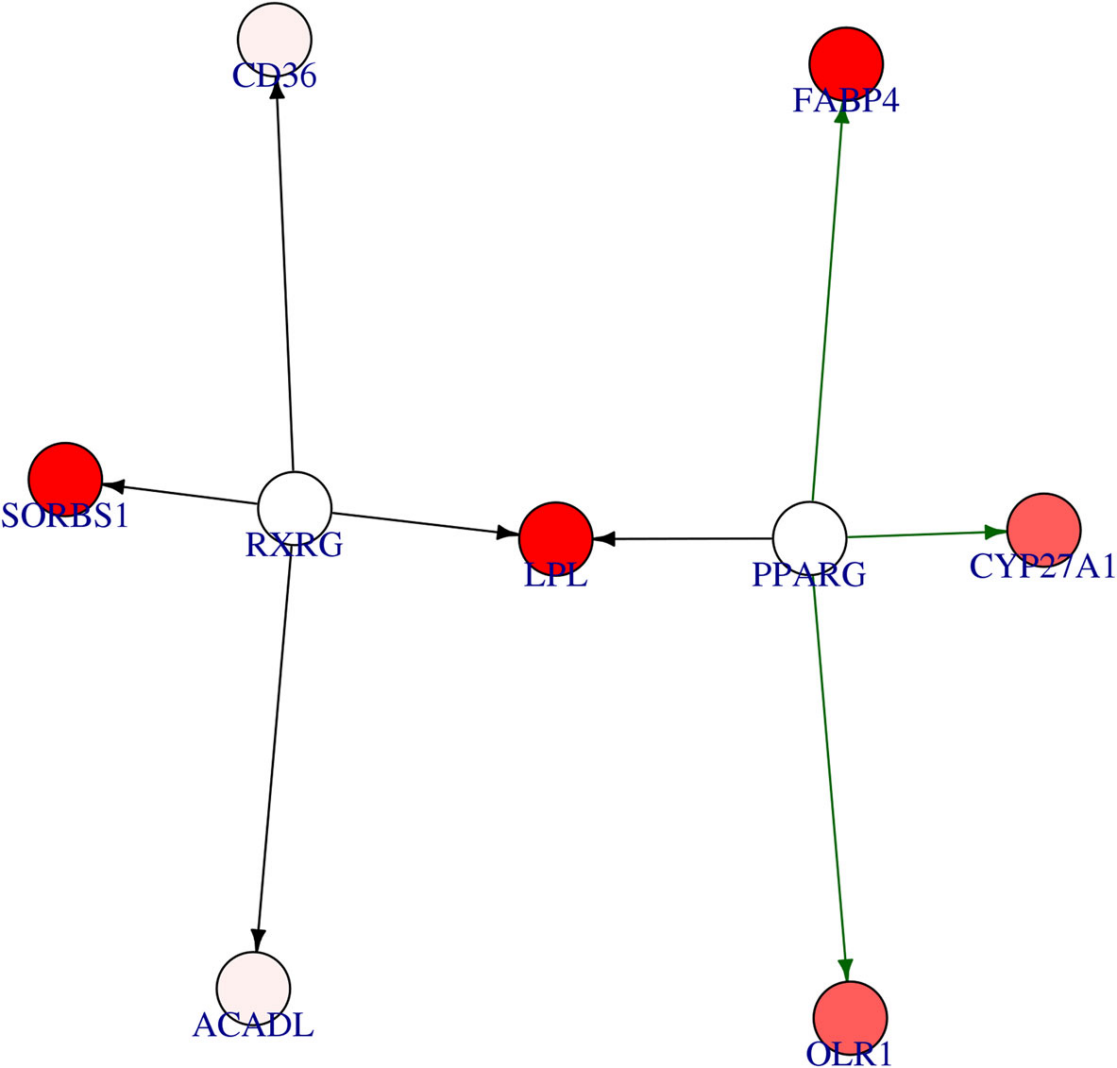
Second active subnetwork from PPI network in tumor samples of 55 patients with LUAD. *DEsubs* R package identified active subnetworks by using q-values of DEGs determined at previous step. Red graduation in nodes indicate the q-value degree, the edge width indicates the correlation degree between the respective genes. Green or red color in edges indicates the positive or negative correlation respectively

**Fig. 10:**
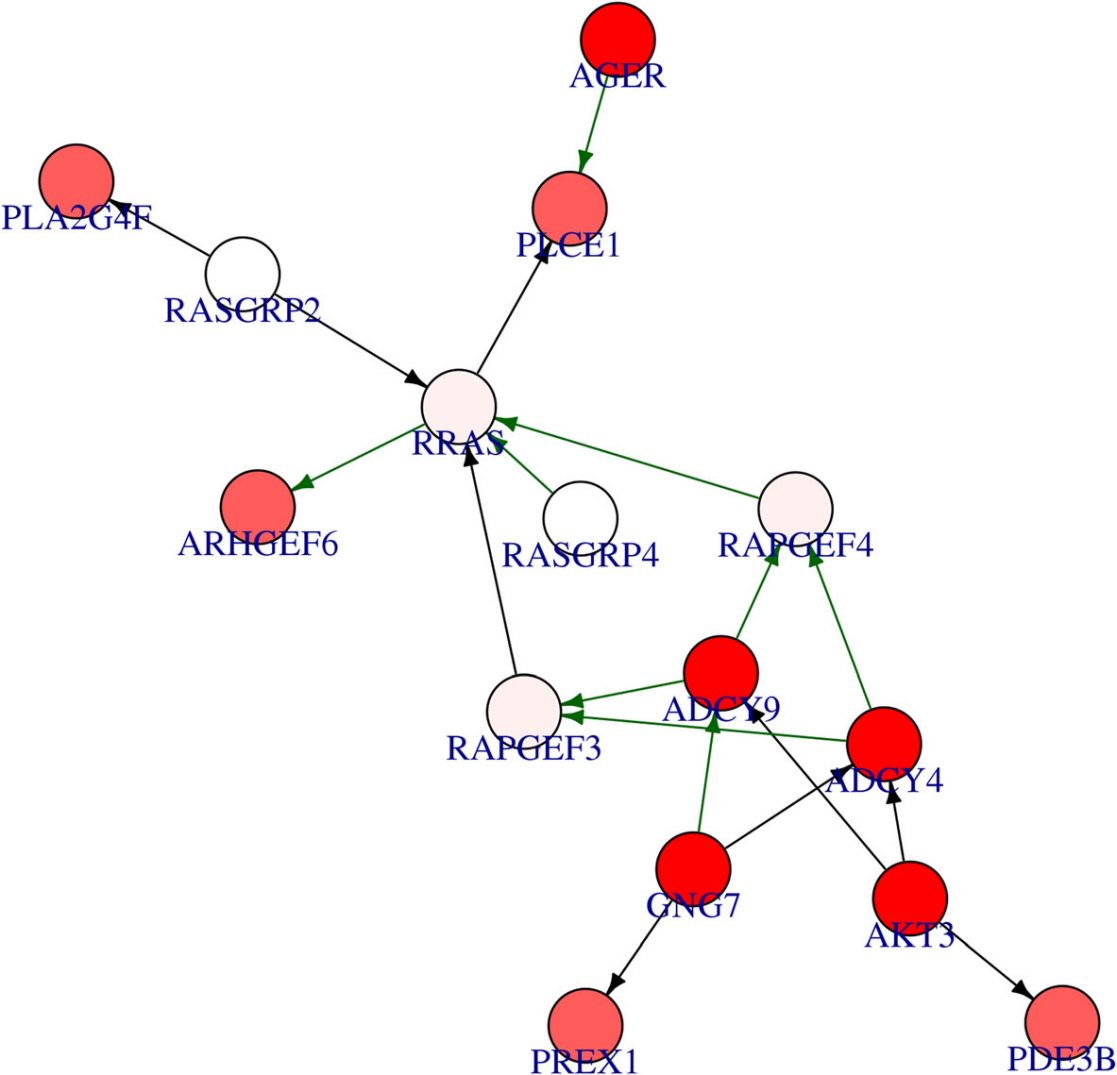
Third active subnetwork from PPI network in tumor samples of 55 patients with LUAD. *DEsubs* R package identified active subnetworks by using q-values of DEGs determined at previous step. Red graduation in nodes indicate the q-value degree, the edge width indicates the correlation degree between the respective genes. Green or red color in edges indicates the positive or negative correlation respectively

**Fig. 11:**
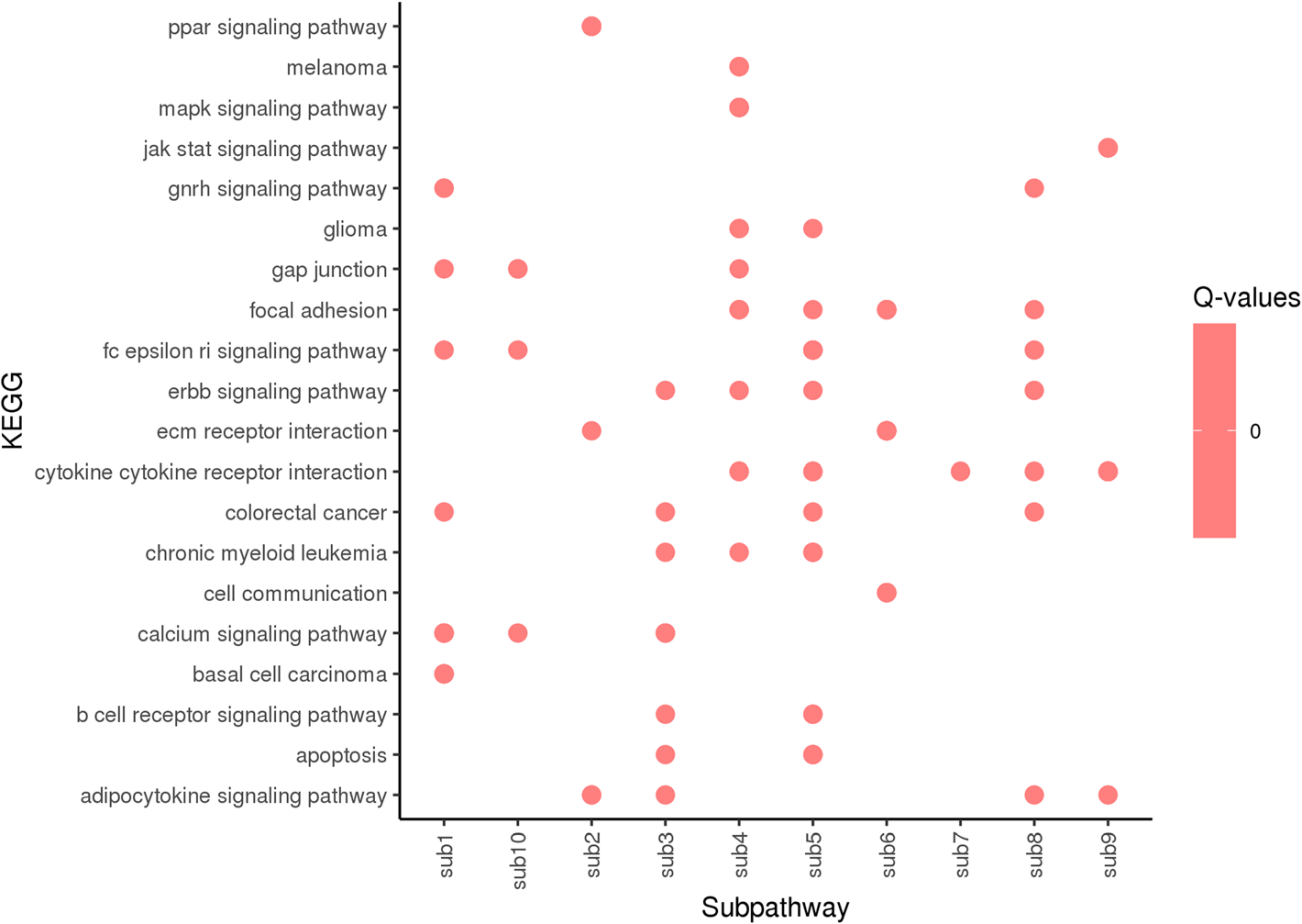
KEGG pathways of active subnetwork genes in tumor samples of 55 patients with LUAD

The output of Differentially Expression Analysis (DEA) containing differentially expressed genes with their Ensembl IDs and adjusted p-values (q-values) were used as input of *DEsubs* R package. The active subnetworks of differentially expressed genes in tumor samples of both 55 LUAD patients were determined by *DEsubs* package and results were represented as graphs at subnetwork and organism levels. *DEsubs* package identified 35 subnetworks including 192 genes, 14 of them including more than three genes, 8 of them including three genes and the others including two genes. In Fig. 7, the top ten significant genes which play a role in determined subnetworks are represented with their q-values. These genes are FABP4, WNT3A, EDNRB, TEK, AGER, EPAS1, ACADL, PDIA4, ANGPT4, KL. In this analysis, 35 subnetworks were determined and the first three subnetworks are presented in Fig. 8, 9 and 10**.** When we look at the subnetworks’ graphs, in subnetwork 1 (Fig. 8), the prominent genes are WNT genes which are members of WNT pathway, a major evolutionary conserved signaling pathway playing role in cell differentiation, cell migration and organogenesis during development and highly related to lung cancer; in subnetwork 3 (Fig. 10), the prominent gene is AKT3 which is one of the AKT family members which play role in tumorigenesis and are modulators of several tumors. The pathways of subnetwork genes are mostly cancer related pathways such as melanoma, glioma, colorectal cancer, chronic myeloid leukemia, basal cell carcinoma, apoptosis, erbb signaling, jak-stat signaling and map kinase signaling pathways (Fig. 11).

### Statistical analysis

**Fig. 12:**
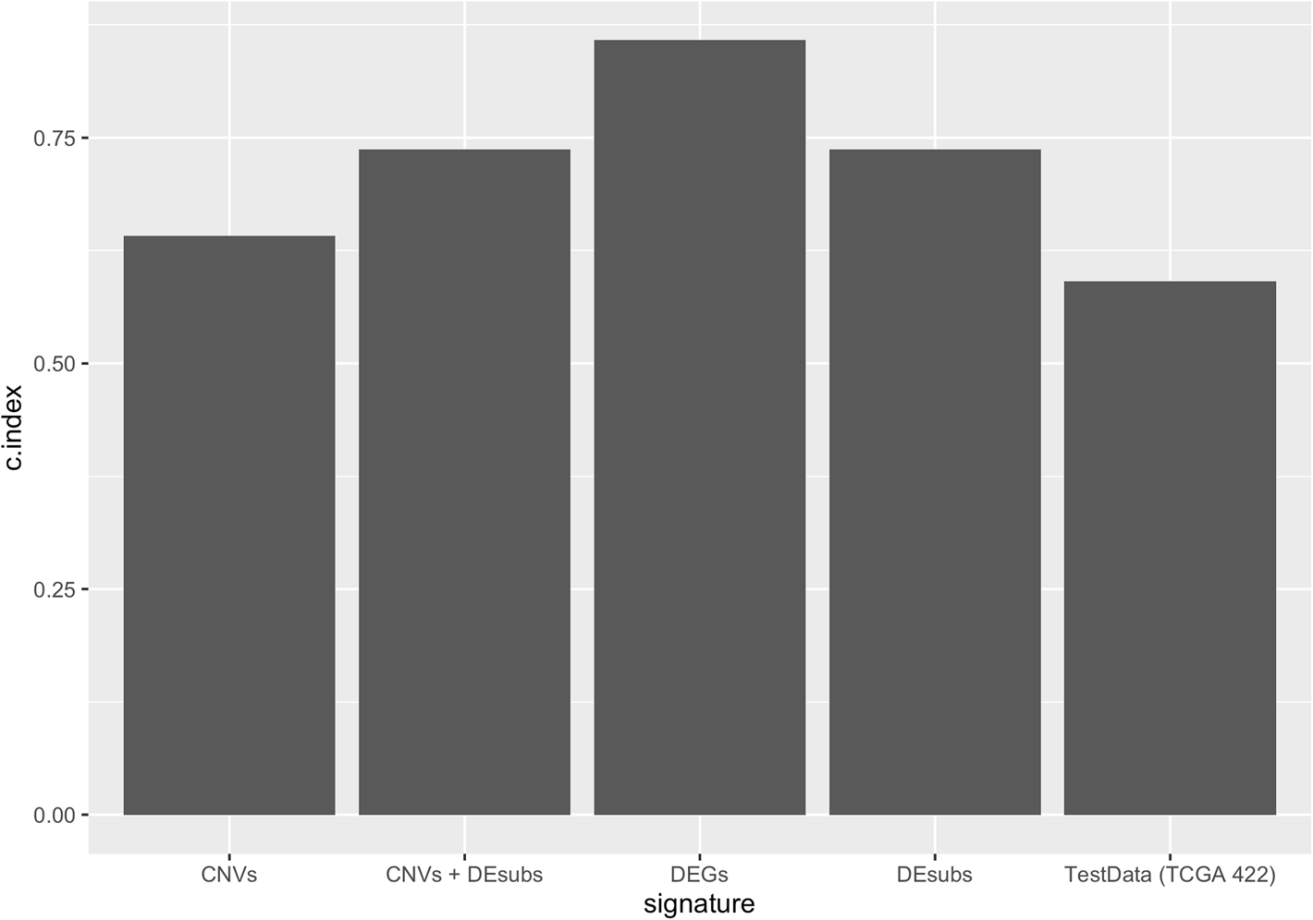
The c-index scores of different gene categories in training data and selected signature in testing data. C-index scores were calculated for different gene categories (DEGs; DEsubs; CNVs; CNVs + DEGs, CNVs + DEsubs; CNVs + DEGs + SNVs; CNVs + DEsubs + SNVs). The categories which have unique signature were used for c-index scoring. CNVs: copy number altered genes, DEGs: Differentially expressed genes, DEsubs: DEGs in active subnetworks

In order to identify a molecular prognosis risk model, the clinical data of all patients in TCGA LUAD project (Table 7) was downloaded by *TCGAbiolinks* R package and separated as training data of 55 LUAD patients who have paired samples for *RNAseq* data and used for gene signature construction; and test data of remaining 422 LUAD patients after removing patients who have missing values in clinical data. Different gene signatures were generated from the genes which have the prognostic ability. The univariate cox regression analysis was performed for significant SNV genes, significant CNV genes, significant DEGs and active subnetwork DEGs in tumor samples of 55 patients with LUAD. There were 38 CNV genes, 463 DEGs and 37 subnetwork DEGs (DEsubs) with prognostic ability after univariate analysis and logRank test (*p* < 0.05). SNV genes did not have significant prognostic ability. Then different data categories (DEGs; DEsubs; CNVs; CNVs + DEGs, CNVs + DEsubs; CNVs + DEGs + SNVs; CNVs + DEsubs + SNVs) were generated by using significant prognostic genes. These data categories underwent the Cox proportional hazards regression with the Lasso penalty and LOOCV. Gene models from different categories were generated by using *glmnet* R package which gives active genes with their coefficients. The genes in the models were DEPTOR, ZBTB16, BCHE, MGLL, MASP2, TNNI2, RAPGEF3, SGK2, MYO1A, CYP24A1, PODXL2, CCNA1 from DEGs category; THRA, RAPGEF3, LAMB2 from DEsubs category; SNX13, AC080080.1, RNMTL1P2, AC080080.2 from CNVs category; THRA, RAPGEF3, LAMB2 from CNVs + DEsubs. The genes in CNVs + DEGs and CNVs + DEGs + SNVs categories were the same with the genes in the DEGs category; the genes in CNVs + DEsubs + SNVs were in the CNVs + DEsubs category. Then, c-index analysis was performed to identify the survival predictive ability of the gene models identified from different categories. The higher c-index score was 0.858 from DEGs gene model (Fig. 12). This gene model (BCHE, CCNA1, CYP24A1, DEPTOR, MASP2, MGLL, MYO1A, PODXL2, RAPGEF3, SGK2, TNNI2, ZBTB16) was chosen as the best candidate prognosis gene signature for LUAD datasets.

**Table 7:** Summary of clinical features of 55 and 510 patients with LUAD

Category	Number	
	55 patients	510 patients
Age at diagnosis (median; range)	66 (42–86)	66 (33–88)
Gender		
Female	33	273
Male	22	237
Tumor stage		
I	28	275
II	12	119
III	12	84
IV	2	25
NA	1	7
Vital status		
Alive	31	326
Dead	24	184

**Fig. 13:**
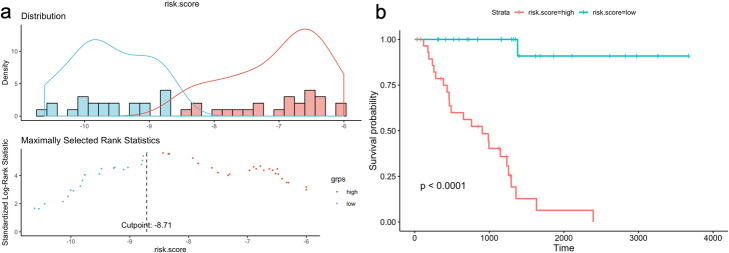
Risk clustering of 55 patients with LUAD and KM survival analysis for training data. **a** Risk score distribution and clustering of patients based on maximally selected standardized log-rank statistic. The patients were clustered into high-risk and low-risk groups based on cutpoint value of the maxstat (maximally selected rank statistics) method. **b** Kaplan-Meier survival plot was generated for high-risk vs low-risk group of 55 patients with LUAD. Time parameter shows the days of overall survival

Multivariate Cox regression analysis was performed for the genes in the chosen 12-gene signature and risk scores of each patient in training data (55 LUAD patients) were calculated by using coefficient values and normalized expression values (log_2_ + 1) in tumor samples. Then the patients were clustered into high-risk and low-risk groups by using maxstat (maximally selected rank statistics) method based on optimal cut-points for numerical variables by using *survminer* R package (Fig. 13a). When we performed Kaplan-Meier (KM) survival analysis to demonstrate the overall survival of risk groups stratified based on gene signature, patients with high-risk score demonstrated poor overall survival (*p* < 0.0001) than those with the low-risk score in training dataset (Fig. 13b).

**Fig. 14:**
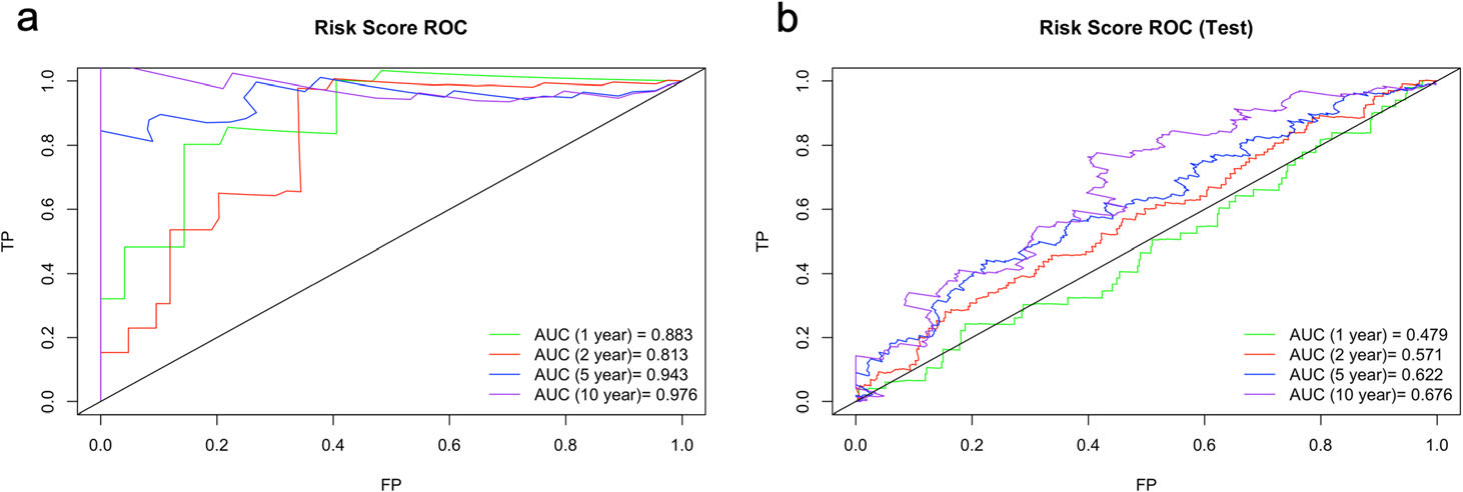
ROC curve analysis for 1, 2, 5 and 10-year survival prediction by the signature. **a** ROC curve analysis for the 12-gene signature in training data. **b** ROC curve analysis for the 12-gene signature in test data. AUC score represents the area under the curve which gives probability of accuracy of the prediction

The ROC curve analysis was performed to compare the sensitivity and specificity of the predictive ability of risk score based on the chosen gene signature. AUC values were 0.883 for 1-year, 0.813 for 2-year, 0.943 for 5-year and 0.976 for 10-year survival prediction (Fig. 14a). These high AUC values showed that the risk scores calculated based on the chosen 12-gene signature can highly predict the overall survival.

**Fig. 15:**
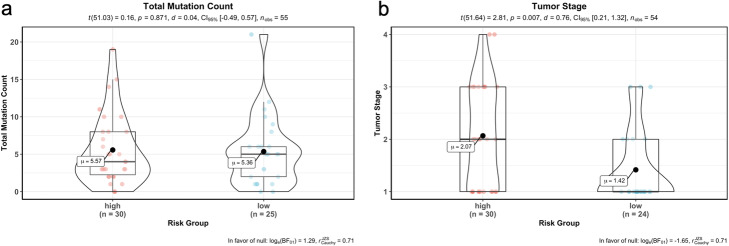
Correlation analysis between risk groups with total mutation count and tumor stage. **a** Total mutation count in tumor samples of training data was not significantly correlated with risk groups. **b** Tumor stage was correlated with risk groups and higher in high-risk group

**Fig. 16:**
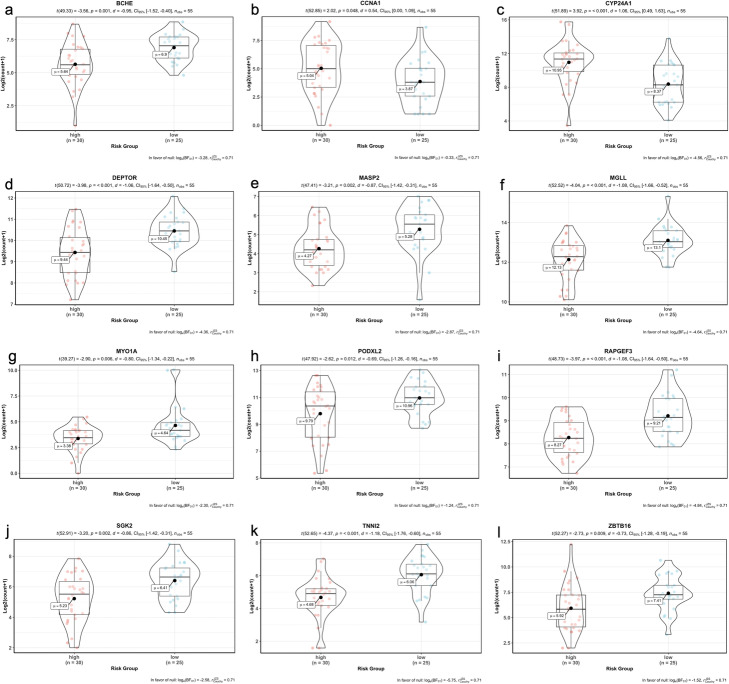
Violin plots showing the expression levels of the 12-signature genes in training data

When we performed the correlation analysis between tumor stages, mutation counts and gene expressions of signature genes, there was a significant difference of tumor stages between risk groups although there was no difference of total SNV mutation count between groups (Fig. 15). However, as expected gene expression levels were significantly different between high-risk and low-risk groups in training data (55 LUAD patients) (Fig. 16). The expression levels of the BCHE, DEPTOR, MASP2, MGLL, MYO1A, PODXL2, RAPGEF3, SGK2, TNNI2, and ZBTB16, genes were lower in the high-risk group while the expression levels of the CCNA1 and CYP24A1 genes were higher in high-risk group (Fig. 16).

**Fig. 17:**
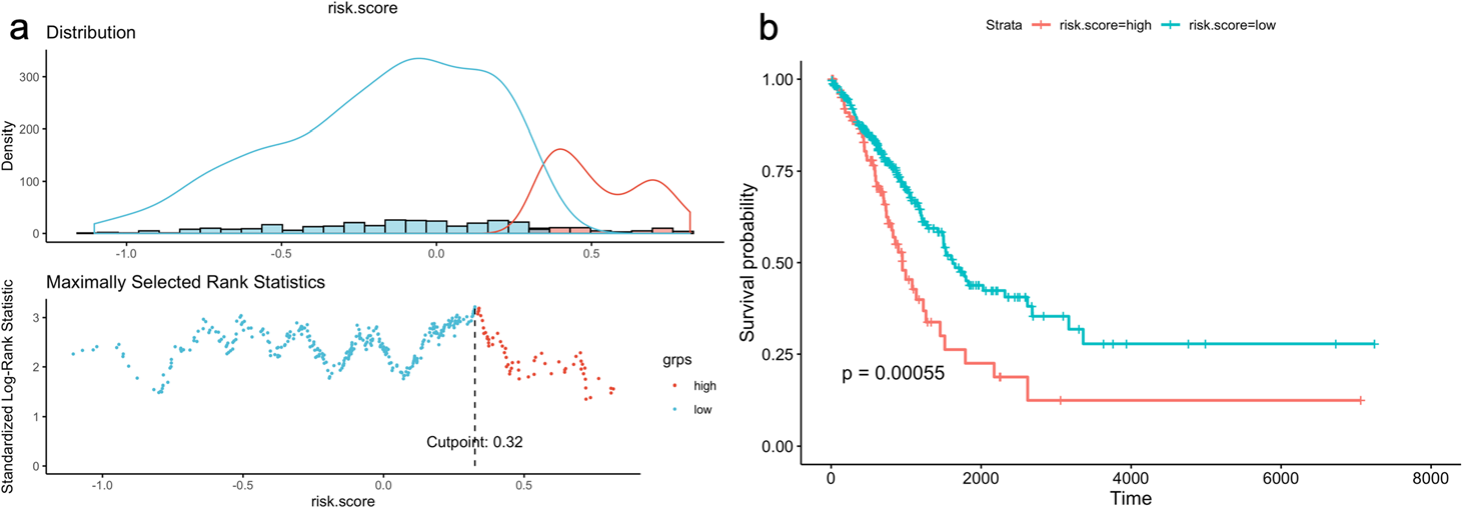
Risk clustering of 422 patients with LUAD and KM survival analysis for test data. **a** Risk score distribution and clustering of patients were performed based on maximally selected standardized log-rank statistic. The patients were clustered into high-risk and low-risk groups based on cutpoint value of the maxstat (maximally selected rank statistics) method. **b** Kaplan-Meier survival plot generated for high-risk vs low-risk group of 422 patients with LUAD. Time parameter shows the days of overall survival

In order to validate our signature, we calculated c-index for the prediction of the overall survival of the 442 TCGA patients with LUAD (test data) and the c-index was 0.591 which is lower than the c-index of training data (Fig. 12). Then, multivariate cox regression analysis was performed for the signature genes in test data. The risk score for each patient was calculated by adding the multiplication of the normalized gene expression level in tumor samples and the multivariate Cox regression coefficient value of each gene in the signature. Patients in the test dataset were divided into high-risk and low-risk groups by using maxstat (maximally selected rank statistics) method from using *survminer* R package (Fig. 17a). Patients in the high-risk group had poor overall survival significantly (*p* < 0.00055) (Fig. 17b). The ROC curve analysis was performed to compare the sensitivity and specificity of the predictive ability of risk score in the test dataset. AUC values were 0.479 for 1-year, 0.571 for 2-year, 0.622 for 5-year and 0.676 for 10-year survival prediction (Fig. 14b). The AUC values of risk scores calculated based on chosen gene signature were very low according to the AUC values of training data. Although the survival predictive ability (c-index) of our gene signature and AUC values of the risk score in test data was low, our 12-gene signature could separate patients into two groups which have a significant overall survival difference (Fig. 17b).

**Fig. 18:**
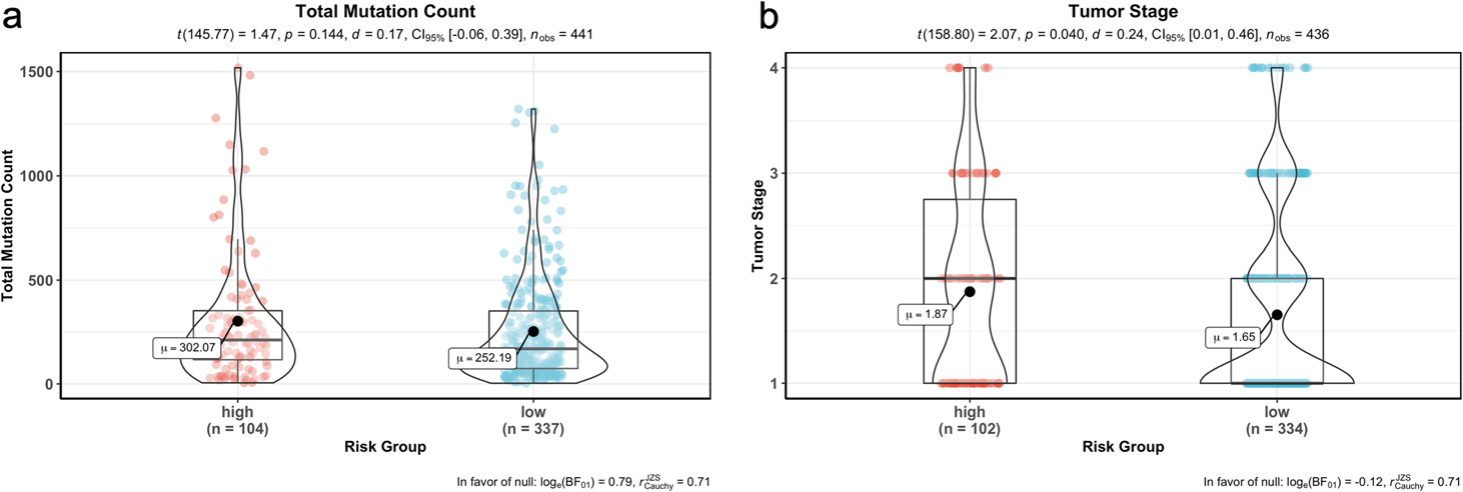
Correlation analysis between risk groups with total mutation count and tumor stage. **a** Total mutation count in tumor samples of test data was not significantly correlated with the risk groups. **b** Tumor stage was correlated with the risk groups of test data and slightly higher in the high-risk group

**Fig. 19:**
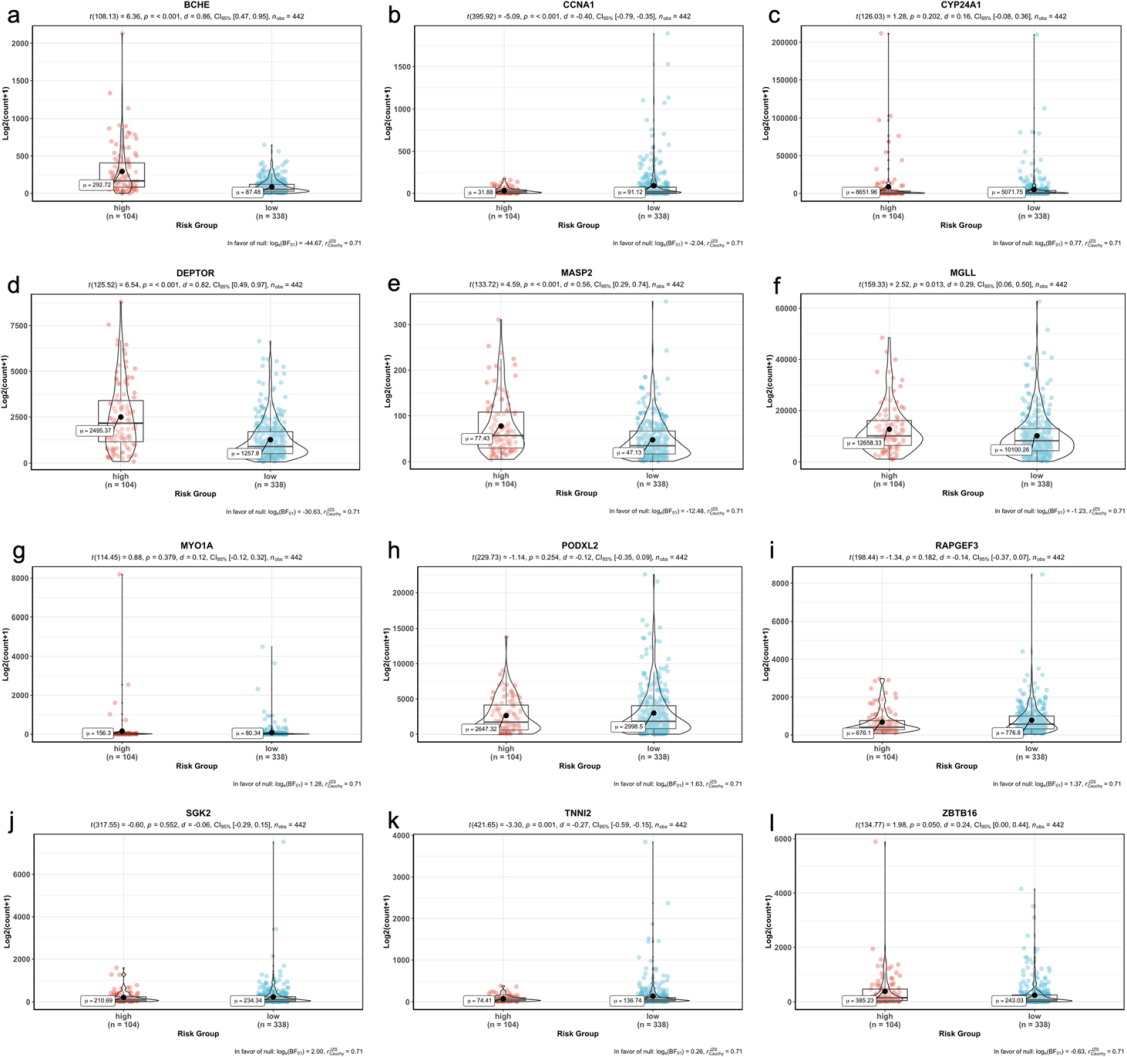
Violin plots showing the expression levels of the 12-signature genes in test data

We performed the correlation analysis between tumor stages, mutation counts and gene expressions of signature genes for test data, there was a slight significant difference of tumor stages between risk groups although there was no difference of total SNV mutation count between groups (Fig. 18). The gene expression levels of 6 signature genes (BCHE, CCNA1, DEPTOR, MASP2, MGLL, TNNI2) were significantly different between the high-risk and low-risk groups however, the gene expression levels of other 6 signature genes (CYP24A1, MYO1A, PODXL2, RAPGEF3, SGK2, ZBTB16) do not have significant difference in test data. The expression levels of the CCNA1 and TNNI2 genes were lower in the high-risk group while the expression levels of the BCHE, DEPTOR, MASP2 and MGLL genes were higher in the high-risk group (Fig. 19).

## Discussion

Lung adenocarcinoma (LUAD) is the most common form of lung cancer which is the most common cancer and responsible for the largest number of deaths worldwide. In order to characterize genomic and transcriptomic abnormalities of lung cancer and to determine the clinical status of patients, integrative analysis have been performed by using different types of molecular data. Recently, prognosis risk signatures have been generated to cluster patients with lung adenocarcinoma. However, mostly gene expression data has been used for this purpose. In this study, we performed an integrative analysis by using level-3 data of SNVs, CNVs and RNAseq data of patients with lung adenocarcinoma in TCGA project. We aimed to identify genomic and transcriptomic abnormalities that might be used to generate a molecular signature. We determined the significantly mutated genes; amplified and deleted genes; and differentially expressed genes (DEGs) significantly and their active subnetworks by using R packages. Then we performed univariate and multivariate Cox Proportional Hazards Regression (CPHR) analysis with LOOCV and the Lasso penalty to identify predictor genes on survival time of patients with lung adenocarcinoma.

Firstly, we identified 6 and 82 mutated genes which are candidate driver genes in tumor samples of 55 LUAD patients and those of 510 LUAD patients, respectively. KRAS and EGFR oncogenes with TP53, STK11, RB1 and MGA tumor suppressors were mutated significantly in the small cohort of patients. The mutated 82 genes of a large cohort of patients include the 6 genes above and also previously identified lung adenocarcinoma related genes such as KRAS, TP53, STK11, RB1, NF1, RMB10, BRAF, KEAP1, CDKN2A, SETD2, ARID1A, SMARCA4 and MGA [5]; EGFR and ERBB2 [4, 5]; and PIK3CA [5, 6]. Besides, MAP2K1 and MAP2K4 mutations can be related with MAPK pathway activity as identified in the TCGA lung adenocarcinoma original article [5]. Loss-of-function MGA mutations with MYC amplification in lung adenocarcinoma have been newly described [5] and MGA gene was identified by *SomInaClust* analysis in our study. MGA, encodes MAX gene-associated protein which is a MYC-interacting transcription factor and antagonizes the transcriptional regulation of MYC involved in cancer processes [14].

We identified amplified and deleted genes which have copy number variations in tumor samples of patients with lung adenocarcinoma. We identified significant copy number altered genes which play role in immune system pathways, metabolism pathways with small cell lung cancer pathway and molecular mechanism of cancer pathway. We analyzed differentially gene expression in tumor samples compared to paired normal samples of 55 patients with lung adenocarcinoma and 3575 genes were dysregulated more than two-fold, significantly (q-value < 0.01). The upregulated genes mostly play role in cell cycle and proliferation pathways such as G2/M damage checkpoint regulation, cell cycle control of chromosomal replication, ATM signaling, hereditary breast cancer signaling, bladder cancer signaling and HIF1 signaling pathways. The downregulated genes play role in cAMP-mediated signaling, g-protein coupled receptor signaling, Gαi signaling and other immune system pathways such as complement system, granulocyte/agranulocyte adhesion and diapedesis, dendritic cell maturation and T helper cell differentiation. Then we determined the differentially expressed genes (DEGs) in active subnetworks of PPI network in tumor samples and we identified 192 DEGs in 35 subnetworks. These genes play role in mostly cancer related pathways such as melanoma, glioma, colorectal cancer, chronic myeloid leukemia, basal cell carcinoma, apoptosis, erbb signaling, jak-stat signaling and map kinase signaling pathways (Fig. 11).

We integrated the significant SNVs, CNVs, DEGs and DEGs in active subnetworks by performing multivariate Cox Proportional Hazards Regression (CPHR) analysis with LOOCV and the Lasso penalty after univariate CPHR, we determined a 12-gene expression signature (BCHE, CCNA1, CYP24A1, DEPTOR, MASP2, MGLL, MYO1A, PODXL2, RAPGEF3, SGK2, TNNI2, ZBTB16) which has 0.858 and 0.591 c-index score for training and test data, respectively. Moreover, this 12-gene expression signature had 0.883, 0.813, 0.943 and 0.976 AUC values for 1, 2, 5 and 10-year survival prediction, respectively, for training data. Same 12-gene expression signature had 0.479, 0.571, 0.622 and 0.676 AUC values for 1, 2, 5 and 10-year survival prediction, respectively, for test data. We clustered the patients for both training and test analysis, into the high-risk and low-risk group based on risk scores calculated by using expression levels and multivariate CPHR coefficients of 12 genes in the signature. Kaplan-Meier survival analysis showed highly significant overall survival difference between the high-risk and the low-risk groups for both training data (*p* < 0.0001) and test data (*p* = 0.00055).

All genes in the 12-gene signature are cancer-related and play role in lung cancer pathways which are the candidates of molecular targeting. BCHE (Butyryl cholinesterase) activity in lung adenocarcinoma is less than in the adjacent non-cancerous tissue [15]; and BCHE is one of two potential diagnostic markers in plasma/serum for non-small cell lung cancer [16]. CCNA1 (Cyclin A1) is a cell cycle regulator protein and was down-regulated in non-small cell lung cancer and CCNA1 promoter was hypermethylated in 70% of lung tumors which has wild-type p53, but was not methylated in cells with mutant p53 [17]. CCNA1 plays a role in p53-mediated G2 cell cycle arrest and apoptosis in non-small cell lung cancer cells and upregulation of cyclin A1 resulted in apoptosis [18]. However, Cho et al. determined that knock-down of CCNA1 using siRNA, induced apoptosis in non-small cell lung cancer cells [19]. CYP24A1 expression level was highly increased in lung adenocarcinoma compared to normal lung tissue samples and CYP24A1 overexpression was associated with poorer survival, increased cell growth and invasion, and increased RAS protein expression in lung adenocarcinoma [20–23]. Knockdown of CYP24A1 significantly decreased cell proliferation resulted in tumor growth delay and smaller tumor size with decreased RAS protein level, thus reducing phosphorylated AKT [21]. DEPTOR (DEP domain-containing mTOR-interacting protein), a natural mTOR inhibitor, was downregulated by activation of EGFR signaling. EGFR inhibition by Gefitinib resulted DEPTOR accumulation. DEPTOR inhibited proliferation, migration, invasion and the tumor growth of lung adenocarcinoma. DEPTOR induction inhibited EGFR mediated tumor progression [24]. DEPTOR depletion can induce EMT in cancer cells and DEPTOR plays a critical role in EMT regulation by BMK1 [25]. DEPTOR was also identified as one of the 77 clinically relevant predictive biomarker at TGFβ-EMT signature generated by microarray analysis of TGFβ-1 treated non-small cell lung cancer cells. TGFβ-EMT gene signature could predicted overall survival and metastasis-free survival in lung adenocarcinoma [26]. MASP-2 (Mannan-binding lectin-associated serine protease 2) is a plasma protein involved in lectin pathway of complement system which promotes cell differentiation, proliferation, migration and reduced apoptosis. Complement activation in the tumor microenvironment enhances tumor growth and increases metastasis [27]. High MASP-2 levels concentration in serum significantly correlated with recurrent cancer disease and with poor survival, thus the MASP-2 level had an independent prognostic value in the patients [28]. MBL/MASP complex activity was significantly increased in patients with colorectal cancer, too [29]. MGLL (Monoglyceride lipase) is highly expressed in aggressive human cancer cells and primary tumors, where it regulates a fatty acid network enriched in oncogenic signaling lipids that promotes migration, invasion, survival, and in-vivo tumor growth [30]. MGLL expression was significantly reduced in the majority of primary human lung cancers and primary colorectal cancers compared to normal tissues [31, 32]. MGLL suppressed colony formation in tumor cell lines and knockdown of MGLL resulted in increased Akt phosphorylation. MGLL plays a negative regulatory role in phosphatidylinositol-3 kinase/Akt signaling and tumor cell growth [32]. MGLL knock-out mice exhibited a higher incidence of neoplasia in lung [31]. MYO1A (Myosin I a) expression was higher in ever smokers than in never smokers [33]. MYO1A had mutations and promoter hypermethylation in patients with colorectal cancer and gastric tumors; therefore, lower levels of MYO1A expression was associated with faster tumor progress and poor prognosis [34, 35]. Podocalyxin is an anti-adhesive transmembrane protein played role in the development of more aggressive breast and prostate cancer [36, 37]. Podocalyxin (including PODXL1, PODXL2 and PODXL3) induction resulted in altered migration and invasion, increased MMP expression with increased MAPK and PI3K activity through forming a complex with Ezrin protein, in breast and prostate cancer [38]. Mammalian exchange protein directly activated by cAMP isoform 1 (EPAC1), encoded by RAPGEF3 gene, acts as guanine exchange factor for Ras-like Rap small GTPases [39]. EPAC1 expression was lower in lung cancer tissue compared to expression in normal specimens and associated with the degree malignancy and lymph-node metastasis [40]. SGK is one of three isoforms of the serum glucocorticoid regulated kinase family of serine/threonine kinases. SGK2 expression was upregulated in hepatocellular carcinoma and its downregulation inhibits cell migration and invasion [41]. Expression level of SGK1 was higher in squamous cell lung cancer and correlated with high grade tumors, tumors size and clinical stage [42]. Protein and mRNA expression of cardiac troponin I (TNNI3) were abnormally detected in non-small cell lung cancer tissues, lung adenocarcinoma cell line and lung squamous cell carcinoma cases while there was negative staining for TNNI3 in non-cancer lung tissues [43]. ZBTB16 (zinc finger and BTB domain containing 16), also known as the promyelocytic leukemia zinc finger protein (PLZF), was down-regulated in lymph node adenocarcinoma metastases and NSCLC samples by hypermethylation in the promoter region [44, 45]. Overexpression of ZBTB16 in lung cancer cell lines inhibited proliferation and increased apoptosis while the depletion of cytoplasmic PLZF was correlated with the high tumor grade, lymph node metastasis, the higher tumor stage and the shorter overall survival [44, 45]. ZBTB16 was also down-regulated in never smoker patients with lung adenocarcinoma [46] and non-small cell lung cancer high-metastatic cell line compared with the low-metastatic cell line [47].

Although the 12-gene signature had low AUC values which means that this 12-signature is not the optimal prediction model, it can be used to cluster patients with LUAD into two risk groups. We could test the signature for different lung adenocarcinoma datasets and check AUC values for them, too. The power of these types of signatures can be increased by performing signature generation from larger cohorts or adding different types of data in order to increase the prediction potential. Although we generated different gene categories to integrate genomic and transcriptomic variations for prognostic risk prediction, DEGs had dominance over genomic alterations. This can be due to the fact that genomic alterations work as promoters which give rise to differential gene expression and this altered gene expression profile determined the new fate of the cell. Therefore, we need integration models for different types of biological data which are not independent of each other. We need also new models for patient-based analysis and/or integration of different data types.

## Conclusions

In this study we analyzed the significant SNVs, CNVs and DEGs in active subnetworks, which have impact on overall survival of patients with lung adenocarcinoma in the TCGA project. We determined 12-genes (BCHE, CCNA1, CYP24A1, DEPTOR, MASP2, MGLL, MYO1A, PODXL2, RAPGEF3, SGK2, TNNI2, ZBTB16) which are highly cancer or lung adenocarcinoma related. These 12 genes are candidates to be used as molecular signature for prediction of overall survival-based risk group of patients with lung adenocarcinoma. These genes can be used to cluster patients and determine the best candidates of drugs for the patient clusters which have different molecular nature. These genes also have potential for targeted cancer therapy of patients with lung adenocarcinoma.

## Methods

### Data

Simple Nucleotide Variation, Transcriptome Profiling, Copy Number Variation and Clinical data of both 55 LUAD patients who have paired (both normal and tumor samples) RNAseq data and of 510 patients who have all four types of data (among all patients in LUAD project) was downloaded separately from TCGA harmonized database by using R/Bioconductor *TCGAbiolinks* package [48]. We analyzed the genomic alteration data including Simple Nucleotide Variations, Copy Number Variations; and transcriptomic variations from RNAseq data, processed using the reference of hg38; and clinical data of LUAD patients (Table 7).

### Identification of the significant simple nucleotide variations

The Mutation Annotation Format (maf) file contained somatic mutations of all patients in TCGA LUAD project, was downloaded using *TCGAbiolinks* package. The other R/Bioconductor package, *maftools* [49], were used to subset original maf file by tumor sample barcodes of patients of interest. *Maftools* package also summarizes the mutations and represents as summary plot and oncoplot. Significantly mutated genes divided into two groups, oncogene (OG) or tumor suppressor gene (TSG), among tumor samples of 55 and 510 patients were identified separately by using *SomInaClust* R package [50]. *SomInaClust* works on the basic assumption that important genes in tumor samples have clustered on sequence and high number of inactivating mutations because of the selective pressure during tumorigenesis. Based on this assumption, oncogenes have clustered mutations, while tumor suppressors have inactivating (protein truncating) mutations. *SomInaClust* uses a reference step in which background mutation rate and hot spots are determined for genes existing in reference mutation database such as the COSMIC database (v88) [51].

### Identification of the significant copy number variations

The CNV dataset for primary solid tumor samples of patients with LUAD, generated by Affymetrix Genome-Wide Human SNP Array 6.0 platform, was downloaded using *TCGAbiolinks* package. The significant aberrant genomic regions in tumor samples of 55 and 510 patients were identified separately by R/Bioconductor *GAIA* package [52]. NCBI IDs and HUGO symbols of the genes with differential copy number were determined using *biomaRt* R package [53].

### Differential expression analysis (DEA)

The Transcriptome Profiling data in mRNA expression level (as unnormalized HTSeq raw counts) of 55 LUAD patients who have paired samples was downloaded by *TCGABiolinks* package. Differentially expressed genes were determined with FDR adjusted *p*-values (q-values) in tumor samples (TP) according to normal samples (NT) of 55 LUAD patients by *limma-voom* method using *limma* [54] and *edgeR* [55] R/Bioconductor packages. NCBI IDs and HUGO symbols of the differentially expressed genes determined by the *biomaRt* R package.

### Active subnetwork and pathway analysis

We identified the active subnetworks of differentially expressed genes in tumor samples of 55 LUAD patients using R/Bioconductor *DEsubs* package [56]. The output of *limma* package containing differentially expressed genes with their Ensembl IDs and FDR adjusted *p*-values (q-values) were used as input of *DEsubs* package. *DEsubs* package determines and represents the active subnetworks with their graphs both at subnetwork and pathway levels.

### Statistical analysis

Clinical data of 55 and 510 patients was downloaded from TCGA database using the *TCGAbiolinks* package. Univariate Cox Proportional Hazards Regression analysis [57] and logRank test [58] were performed using *survival* R package [59] for the significant SNV containing genes, the significant CNV containing genes, the DEGs and the active subnetwork genes to identify genes with prognostic ability. For the genes with prognostic ability (p value < 0.05), multivariate Cox proportional hazards model with LOOCV and the Lasso penalty was used to identify the best gene signature among different combinations of molecular levels (SNV genes, CNV genes, DEGs and active subnetwork genes) by using *glmnet* R package [60]. Concordance index (c-index) was performed using *pec* R package [61] to validate the predictive ability of different gene signatures. The larger c-index is used to determine the gene signature which has more accurate predictive ability. Multivariate cox proportional regression analysis was performed using *survival* R package for genes of selected signature and the risk score of each patient was calculated using coefficient and expression values of the genes. Then, patients were clustered into the high-risk group and the low-risk group and Kaplan-Meier (KM) survival curves [62] were generated using *survminer* R package [63] to demonstrate the overall survival of risk groups stratified based on gene signature. ROC curve analysis [64] was also performed for risk scores calculated based on selected gene signature by using *survivalROC* R package.

Significant differences in the tumor stages, the mutation counts and the expression levels of patients in the high-risk and low-risk groups were identified using *ggstatsplot* R package [65]. In order to validate the prognosis risk signature, the risk scores of 442 TCGA patients with LUAD were calculated using the expression values of the gene signature and their coefficient values from multivariate Cox proportional regression analysis. Similarly, 442 patients (after exclusion of 55 and other patients with missing data from 510 patients) were clustered into high-risk and low-risk groups and the overall survival difference between the two groups of patients was assessed by KM survival curve. Significance level used for identification of genes containing copy number variations and differentially expressed genes, was 0.01 for FDR corrected q-value. Significance level was 0.05 for FDR corrected *p* values (q value) for identification of genes containing the significant single nucleotide variations; and was 0.05 for *p*-values for the active subnetwork and the pathway analysis, and for all the statistical analysis.

## Data Availability

The datasets supporting the conclusions of this article are publicly available and can be downloaded from TCGA data portal (https://portal.gdc.cancer.gov) or by using *TCGAbiolinks* R package. The R code used in this study is available upon request.

## Abbreviations

AUC: Area Under Curve
C-index: Concordance index
CNVs: Copy Number Variations
CPHR: Cox Proportional Hazards Regression
DEGs: Differentially Expressed Genes
DEsubs: DEGs in active subnetworks
EMT: Epithelial-Mesenchymal Transition
FDR: False Discovery Rate
GEO: Gene Omnibus Database
HGNC: HUGO Gene Nomenclature Committee
KM: Kaplan-Meier
logFC: Log_2_ Fold Change
LOOCV: Leave-One-Out Cross Validation
LUAD: Lung adenocarcinoma
miRNA: MicroRNA
NSCLC: Non-Small Cell Lung Cancer
OG: Oncogene
PPI: Protein-Protein Interaction
RNA-seq: RNA sequence
ROC curve: Receiver Operating Characteristic curve
siRNA: Silencing RNA
SNVs: Single Nucleotide Variations
TCGA: The Cancer Genome Atlas
TSG: Tumor Suppressor Gene
